# Pseudoknots in RNA folding landscapes

**DOI:** 10.1093/bioinformatics/btv572

**Published:** 2015-10-01

**Authors:** Marcel Kucharík, Ivo L. Hofacker, Peter F. Stadler, Jing Qin

**Affiliations:** 1 ^1^Institute for Theoretical Chemistry,; 2 ^2^Research Group BCB, Faculty of Computer Science, University of Vienna, Austria,; 3 ^3^RTH, University of Copenhagen, Frederiksberg, Denmark,; 4 ^4^Department of Computer Science & IZBI & iDiv & LIFE, Leipzig University,; 5 ^5^Max Planck Institute for Mathematics in the Sciences,; 6 ^6^Fraunhofer Institute IZI, Leipzig, Germany,; 7 ^7^Santa Fe Institute, Santa Fe, NM 87501, USA and; 8 ^8^IMADA, University of Southern Denmark, Campusvej 55, Odense, Denmark

## Abstract

**Motivation:** The function of an RNA molecule is not only linked to its native structure, which is usually taken to be the ground state of its folding landscape, but also in many cases crucially depends on the details of the folding pathways such as stable folding intermediates or the timing of the folding process itself. To model and understand these processes, it is necessary to go beyond ground state structures. The study of rugged RNA folding landscapes holds the key to answer these questions. Efficient coarse-graining methods are required to reduce the intractably vast energy landscapes into condensed representations such as barrier trees or basin hopping graphs **(**BHG) that convey an approximate but comprehensive picture of the folding kinetics. So far, exact and heuristic coarse-graining methods have been mostly restricted to the pseudoknot-free secondary structures. Pseudoknots, which are common motifs and have been repeatedly hypothesized to play an important role in guiding folding trajectories, were usually excluded.

**Results:** We generalize the BHG framework to include pseudoknotted RNA structures and systematically study the differences in predicted folding behavior depending on whether pseudoknotted structures are allowed to occur as folding intermediates or not. We observe that RNAs with pseudoknotted ground state structures tend to have more pseudoknotted folding intermediates than RNAs with pseudoknot-free ground state structures. The occurrence and influence of pseudoknotted intermediates on the folding pathway, however, appear to depend very strongly on the individual RNAs so that no general rule can be inferred.

**Availability and implementation:** The algorithms described here are implemented in C++ as standalone programs. Its source code and Supplemental material can be freely downloaded from http://www.tbi.univie.ac.at/bhg.html.

**Contact:** qin@bioinf.uni-leipzig.de

**Supplementary information:** Supplementary data are available at *Bioinformatics* online.

## 1 Introduction

Beyond the role as carriers of genetic information, RNA molecules often play much more active roles in regulating gene expression, intracellular transport and even as catalysts (Cech and Steitz, 2014). More often than not, these functions are associated with the RNAs’ ability to undergo specific conformational changes, as is the case for riboswitches. The function of an RNA molecule thus is often poorly described by its ground state structure and instead has to be studied as a dynamic ensemble of structures (Dirks *et al.*, 2004; Onoa and Tinoco, 2004). Quantities of biological interest include folding times, life times of meta-stable states and folding pathways. Riboswitches that control transcription, for example, often function through finely balanced time-scales of transcriptional elongation and formation of a terminator hairpin structure (Barrick and Breaker, 2007). These relevant kinetic parameters can in principle be derived from the folding landscapes.

The most direct way of dealing with the ensemble aspect of an RNA is to enumerate its entire energy landscape. In addition to the list of conformations, the landscape picture emphasizes a notion of adjacency between RNA structures. In most cases, opening or closing of a single base pair is taken to be the elementary operation, and thus as the definition of adjacency between two structures (Flamm *et* *al.*, 2000a). The dynamics of folding is then modeled as a Markov process of transitions between adjacent conformations with transition rates estimated from energy differences using, e.g. the Metropolis rule (Flamm *et* *al.*, 2000a; Smit *et al.*, 2007; Xayaphoummine *et* *al.*, 2007).

Except for very short RNAs, this approach is not feasible in practice because the number of secondary structures grows exponentially with sequence length (Hofacker *et* *al.*, 1996). The dynamic programming algorithms for finding the ground state or evaluating the partition function can be modified to enumerate only the lowest energy states (Wuchty *et* *al.*, 1999). Even so, condensed representations are required to gain insights into the properties of the energy landscapes that are relevant for the definition of folding pathways and the interpretation of folding kinetics. The first representation of this type is a barrier tree with local minima as leafs and saddle points as interior nodes. This notion has been developed independently in different contexts including spin glasses (Klotz and Kobe, 1994; Sibani *et* *al.*, 1999), potential energy surfaces for protein folding (Garstecki *et* *al.*, 1999; Wales, 2011), molecular clusters (Doye *et* *al.*, 1999) and RNA secondary structures (Flamm *et* *al.*, 2000a). The kinetics on the landscape can then be approximated by the Arrhenius law on the barrier tree. However, this abstraction has significant shortcomings. It completely neglects both the entropic information on the size and the shape of the basin surrounding its corresponding local minimum (LM), and the topological information of their relative locations. Wolfinger *et* *al.* (2004) showed that much of the entropic effects can be captured by partitioning the landscape into the basins of LMs. This yields a barrier tree with energy scales in terms of energies of basins rather than structure energies. But, still the barrier tree necessarily ignores the general topology of the landscape since in most cases there are more than one folding pathways between RNA structures.

Kucharík *et* *al.* (2014) introduced the basin hopping graph (BHG) to capture more information regarding adjacency between LMs. Nodes in the BHG are LMs, and two LMs are neighbored only if the direct transition between their corresponding basins are ‘energetically favorable’. The corresponding saddle height is annotated on the edge. In this abstraction, possible folding pathways are represented as sequences of adjacent basins represented by their LMs. The BHG is particularly suitable to describe the ruggedness of RNA folding landscapes and to explain the interconversion between multiple ‘active’ LMs as observed by Solomatin *et* *al.* (2010). Like barrier trees, BHGs can be obtained by complete enumeration for small RNAs. Kucharík *et* *al.* (2014) also developed an efficient and accurate heuristic that makes the approach feasible for RNA molecules with a length up to ∼200 nucleotides (nt).

So far, these techniques are largely restricted to pseudoknot-free secondary structures despite the fact that pseudoknots are crucial for the function of many RNA elements, e.g. ribosomal frame-shifting (Giedroc *et* *al.*, 2000), regulation of translation and splicing (Draper *et* *al.*, 2000), or the binding of small molecules (Gilbert *et* *al.*, 2008; Klein *et* *al.*, 2009; Spitale *et* *al.*, 2009). Large RNAs often feature long-range pseudoknots (Adams *et al.*, 2004; Klein and Ferre-DAmare, 2006; Toor *et* *al.*, 2008) that may play important roles in both biochemical function and mechanical stability (Chen *et* *al.*, 2009). Even though pseudoknots have been considered in the contexts of folding pathways and kinetic mechanisms in particular case studies (Cho *et* *al.*, 2009; Engel *et* *al.*, 2014; Isambert and Siggia, 2000; Roca *et* *al.*, 2015), the energy landscapes of RNAs with pseudoknots and other tertiary contacts have not received much systematic attention. There are several reasons for this state of affairs: (i) detailed thermodynamic and kinetic measurements on pseudoknots are still rare despite recent progress (Liu *et* *al.*, 2010) so that energy models for pseudoknotted RNAs are crude approximations at best; (ii) computational methods for sampling pseudoknotted structures are expensive in terms of both CPU time and memory (Reidys, 2011) and (iii) there are many competing alternative definitions of the space of pseudoknotted structures ranging from small extensions of pseudoknot-free structures to essentially arbitrary matchings (Condon *et al.*, 2004; Lyngso and Pedersen, 2000; Nebel and Weinberg, 2012).

The contribution of this article is two-fold: First, we demonstrate that the BHG can be computed in practice using the so-called 1-structures as its search space as described by the gfold (Reidys *et* *al.*, 2011) algorithm and the work of Bon *et* *al.* (2008). To this end, we propose an efficient sampling algorithm for detecting LMs and we generalize the estimation of direct saddles to structures with pseudoknots. We will see that the inclusion of pseudoknotted structures indeed leads to a significant reduction in saddle heights. Second, we model the folding kinetics as a continuous-time Markov chain on the BHG to investigate the effects of pseudoknotted LMs on the folding kinetics. This contribution is organized as follows: In Section 2.1, we generalize the existing BHG model by taking pseudoknotted structures into consideration. Next, in Section 2.2, we describe the continuous-time Markov chain simulation based on the BHG and the quasi-steady-state (QSS) strategy utilized to reduce the dimension of our model. In Section 3, we present and discuss our experimental results. Section 4 summarizes our findings and suggests directions for future work.

## 2 Methods

### 2.1 BHG of pseudoknotted RNAs

We start with a brief, conceptual description of the BHG of an RNA landscape. Complete formal definitions can be found in Part A of the Supplementary material (SM) and in our previous publication (Kucharík *et* *al.*, 2014). Consider two LMs *x* and *y* and a path *P* connecting them in the landscape. A structure of maximal energy along *P* is called a peak. A saddle point between *x* and *y* is a peak along a particular path from *x* to *y* with minimal possible energy. We say that *P* is a direct path between *x* and *y* if *P* contains a peak *s* such that the energy is non-decreasing along *P* from *x* to *s* and non-increasing from *s* to *y.* A direct path is energetically optimal if its peak is a saddle point between *x* and *y*, i.e. if the direct path is an energetically optimal connection between *x* and *y.* The edges of the BHG correspond exactly to these energetically optimal transitions. A diagram to illustrate these concepts is provided in Supplementary Figure S2.

In Kucharík *et* *al.* (2014), we described an efficient heuristic to estimate the BHG for pseudoknot-free structures. It consists of two independent components: (1) A sample set of LMs within a user-defined energy range above the structure with minimum (free) energy (MFE structure) is produced by simulating gradient walks starting from randomly sampled structures. This step is implemented in the program RNAlocmin. (2) Direct saddle connections between LMs are constructed by a heuristic that iteratively improves initial paths and expands the initial LM set by additional indispensable intermediate LMs. Both construction procedures can be generalized to structures with pseudoknots in a conceptually straightforward manner. On the technical side, however, we encounter non-trivial problems.

**Fig. 1.**
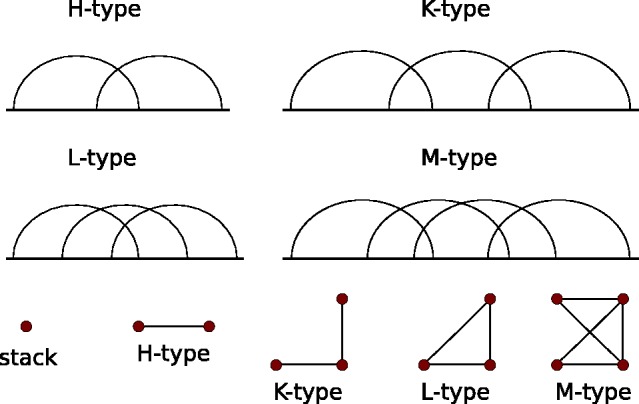
(Top) Four basic types of pseudoknots considered in gfold program. (Bottom) The conflict graph can only have either isolated vertices or four types of components of size >1

Since the inclusion of pseudoknots dramatically enlarges the search space, exhaustive enumeration is not feasible in practice; hence, we have to generalize RNAlocmin for pseudoknotted structures. RNAlocmin works by producing a Boltzmann-weighted sample of initial structures generated by stochastic backtracking. To our best knowledge, the only tool that does Boltzmann sampling of structures with pseudoknots is gfold (Reidys *et* *al.*, 2011). Its sampling space is restricted to a class of pseudoknotted structures which are characterized by the topological genus to be 1 as their ‘elementary’ components and therefore referred to as ‘1-structures’. This class comprises the four basic types of pseudoknots shown in Figure 1—the most common H-type and kissing hairpin (K-type) together with more exotic L-type and M-type pseudoknots. It includes virtually all pseudoknot structures that have been discovered so far (Bon *et* *al.*, 2008). The Boltzmann sampling from 1-structures is computationally demanding. It takes $O(n^{6})$ time to computed the partition function and then $O(n^{5})$ time to sample a single structure of length *n.* This first step is asymptotically optimal. The sampling step could probably be expedited considerably e.g. using the boustrophedon method (Ponty, 2008). In practice, our current implementation is limited to an RNA of length ∼130 nt. In SM Part B, we summarized the technical adaptations that had been made to ensure the consistency of the energy model within our BHG framework.

Gradient walks and connecting paths are also more difficult to implement for pseudoknotted structures. The key issue is to determine whether the insertion of a base pair leads outside the class of 1-structures. The corresponding problem for secondary structures is simple: it suffices to check whether the proposed extra base pair crosses an existing base pair or not. For 1-structures, we construct the conflict graphs whose vertices are the helices. An edge connects two helices whenever they cross. For a 1-structure, its conflict graph consists of isolated vertices and the four types of connected components shown in Figure 1 bottom. Relatively simple manipulations of conflict graphs can be used to decide efficiently whether a particular base pair can be added. For details, we refer to SM Part B.

In order to determine the BHG-adjacency between LMs, we extended the findpath heuristic (Flamm *et* *al.*, 2000b) to compute near optimal folding paths involving pseudoknotted structures. Allowing pseudoknots should always result in lower or equal barrier heights. However, since the accuracy of the findpath heuristic decreases as the landscape grows, its estimation results can in rare cases be slightly worse than the original (pseudoknot-free) findpath. We will return to this point in Section 3.1.

### 2.2 RNA folding kinetics

From a microscopic point of view, the dynamics on an RNA folding landscape can be described by a continuous-time Markov process with infinitesimal generator $R=(r_{yx})$ (Flamm *et* *al.*, 2000a). The transition rate *r_yx_* from a secondary structure *x* to *y* is non-zero only if *x* and *y* are adjacent, i.e. if they differ by adding/removing a single base pair. Typically, the Metropolis rule, $r_{yx}=r_{0}\min\{\exp\{-(f(y)-f(x))/RT\},1\}$, is used to assign microscopic rates. Here, *f* evaluates the (free) energy of *x*, *R* is the universal gas constant, *T* is the absolute ambient temperature and *r*_0_ is a parameter used to gauge the time axis from experimental data. Here we simply use $r_{0}=1$, implicitly defining our time unit. On the BHG, we use the Arrhenius approximation. For two adjacent LMs *x* and *y* with saddle height *S*(*x*, *y*) between them we set

(1) $$r_{xy}=\exp\left(-(\text{S}(x,y)-f(y))/RT\right)\;.$$

For all other pairs of LMs, *r_xy_* = 0. Kinetic trajectories are computed by numerically computing the matrix exponential exp(tR). We have shown already in previous work that the Arrhenius formula on BHG is an excellent approximation of the dynamics on all time scales (Kucharík *et* *al.*, 2014). Analogous validation data are given in SM Part C.

The number of LMs in the energy landscape of randomly generated RNA sequences grows roughly as the square root of the total number of structures (Lorenz and Clote, 2011). Most of these LMs, however, contribute only to fast fluctuations because they have narrow basins and low barriers. We therefore adopt the QSS strategy (Rao and Arkin, 2003; Schuster and Schuster, 1989) to reduce our model complexity. The key idea is to reduce the dimension of the model by removing intermediate QSS and to update the transition rates between the remaining states if correlated. To this end, one assumes that population of a QSS remains unchanged over the time of the simulation. In general, the *a priori* identification of QSS intermediates is a hard problem. Here, however, we can simply use the degree of LMs in the BHG: LMs with low degree are typically intermediates of quick folding pathways between LM with primary function and their population stays extremely low during whole simulation. Further technical details can be found in SM Part D. Throughout this contribution, the state spaces of the examples are pruned to at most 5000 LMs. For clarity, an LM is included in a visualization only if its population exceeds 7% at some time during the simulation.

## 3 Results and discussion

### 3.1 Pseudoknotted LMs’ role in folding

We first analyze the composition of the LMs in the ‘lower’ part of the energy landscapes of RNA molecules, which we take here as structures within 10 kcal/mol above the minimal free energy of the whole landscape. We contrast RNAs with pseudoknots in their ground state selected from Pseudobase++, (Han *et* *al.*, 2002; Taufer *et* *al.*, 2009) and pseudoknot-free structures from the RNA STRAND database (Andronescu *et* *al.*, 2008). In addition, we select the molecules such that their MFE structures predicted by gfold have both sensitivity and PPV beyond 80%, so that effects caused by the prediction software can be limited. A statistic summary of the selected RNAs is provided in Supplementary Table S2 Part E.

In Supplementary Table S3, we report the composition of the LMs obtained by gradient walks starting from gfold-sampled structures. Analogous result of these sampled structures is summarized in Supplementary Table S4.

In our test set, LMs with pseudoknots occupy on average about 75% of LMs included in the BHG if the ground states contains pseudoknots. For RNAs with pseudoknot-free ground states, only about 35% of the nodes in the BHG contains pseudoknots. These data suggest that pseudoknotted LMs can dominate the BHG only if the ground state is also pseudoknotted. Furthermore, it follows from the pseudoknot energy model of gfold (SM Part B) with its large penalties for pseudoknots that a gradient walk starting from a pseudoknot-free structure cannot lead to a pseudoknotted LM. Gradient walks starting from pseudoknotted structures preferrentially terminate in pseudoknot-free or H-type pseudoknotted LMs due to the even larger penalties assigned to the more complex pseudoknot classes K, L and M.

General combinatorial arguments show that for n→∞ almost all structures contain pseudoknots (Saule *et* *al.*, 2011). The energy model, however, ensures that they are fairly rare among the stable structures at the length scales of n≈100…300 nt that we can investigate computationally and that are of most direct interest for experimental studies of RNA folding kinetics. Furthermore, folding is typically dominated by local rearrangements, so that conclusions drawn for moderate-size domains are likely to carry over to *most* transitions along the folding pathways of very large RNAs. In other words, even if pseudoknots appear almost certainly somewhere in long RNAs, they are still sparse and most of the local folding at length scales of around 100 nt is still dominated by pseudoknot-free structures.

A central question to ask is ‘What is the role of pseudoknotted LMs in RNA folding pathways?’. One might expect that they help decrease the saddle heights between structures. We therefore consider, for an RNA whose ground state is pseudoknot-free the full BHG${}^{\psi }$ including pseudoknotted LMs and a pruned BHG${}^{{}^{\circ}}$ in which first all pseudoknotted LMs are removed from BHG${}^{\psi }$ and then the BHG-adjacency is recomputed using only pseudoknot-free structures along the paths. This re-evaluation may result in the removal of adjacencies from BHG${}^{\psi }$.

**Fig. 2.**
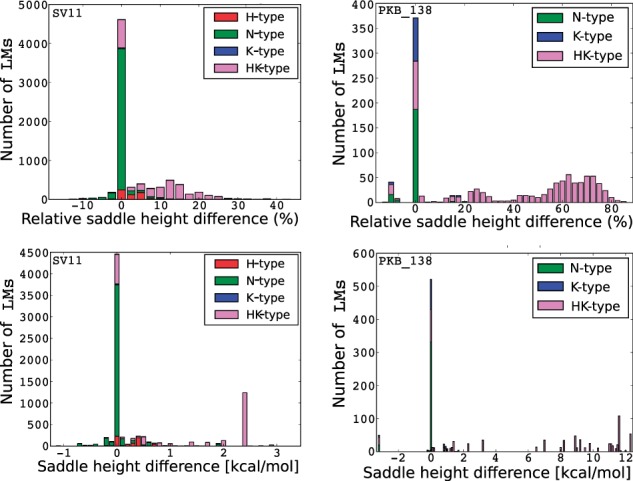
Histograms of saddle height changes between BHG${}^{\psi }$ and BHG${}^{{}^{\circ}}$ for a substrate for SV11 (left) and the tRNA-like pseudoknot of barley stripe mosaic virus PKB_138 (right). (Top left/right) The *x*-axes denote the relative changes (%) between saddle heights of LMs pairs in BHG${}^{\psi }$ and BHG${}^{{}^{\circ}}$ and *y*-axes are the corresponding numbers of pseudoknot-free LMs pairs with such saddle changes. (Bottom left/right) The *x*-axes denote the exact changes (kcal/mol). Colors indicate the pseudoknot types appearing in the energetically optimal paths between LM pairs. Green (N-type) indicates the simulated paths do not contain any pseudoknotted structures. Pink (HK-type) indicates the simulated paths contain pseudoknotted structures of both H-type and K-type. More examples can be found in SM Part F (Color version of this figure is available at *Bioinformatics* online.)

We illustrate in Figure 2 the saddle-height differences between BHG${}^{\psi }$ and BHG${}^{{}^{\circ}}$ for two RNA molecules, a substrate for Qβ replicases (SV11, 115 nt, pseudoknot-free native state—Biebricher and Luce, 1992) and an H-type pseudoknot forming a tRNA-like structure at the 3′end of RNA beta of *barley stripe mosaic virus* (Pseudobase entry PKB_138, 96 nt). See SM Part F for additional examples. Note here, saddle heights between LMs in BHG${}^{{}^{\circ}}$ should never be lower than in BHG${}^{\psi }$. In practice, however, the inclusion of additional LMs during the recomputation of the adjacencies can in rare cases lead to a decrease in estimated saddle heights. In these cases, the saddle heights in BHG${}^{\psi }$ are overestimated due to the heuristic nature of the findpath method. The effect can be reduced by a moderate increase of findpath’s search depth (using the –depth parameter), see SM Part F for further details.

We observe that pseudoknotted LMs help to reduce saddle heights more significantly in the RNAs with pseudoknotted ground states. This is a direct consequence of the large energy penalties associated with pseudoknots, which makes is energetically expensive to nucleate a pseudoknot *directly* from a pseudoknot-free structure without certain detour. For PKB_138, these two types of pseudoknotted LMs help over 42% pairs of LMs to reduce their saddle heights beyond 50%, or up to 12.3 kcal/mol in absolute terms. In the case of SV11, the improvement is not that significant (about 23% of pairs reduce their saddle heights beyond 10% up to 3.4 kcal/mol). Nevertheless, pseudoknotted LMs play an important role in reducing the saddle height between the meta-stable and ground state, see the more detailed discussion in Section 3.2.

We next study the effects of pseudoknotted LMs on the folding kinetics. Here, we restrict ourselves to RNAs with pseudoknot-free ground states since a direct comparison is not possible for pseudoknotted structures. Furthermore, we require that the native structures are well predicted by gfold (both sensitivity and PPV beyond 80%).

**Fig. 3.**
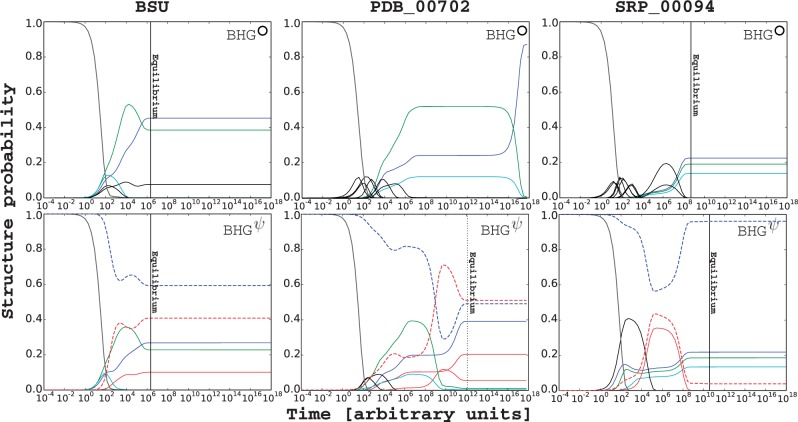
Folding kinetics comparison between BHG${}^{{}^{\circ}}$ (top) and BHG${}^{\psi }$ (bottom) of the preQ_1_ riboswitch (Bsu, left), the Ribosomal RNA from *E.coli* (PDB_00702, middle), and the signal recognition particle RNA (SRP_00094, right). The process was started in the open chain structure and run until convergence to the thermodynamic equilibrium distribution except the case of PDB_00702 on BHG${}^{{}^{\circ}}$ where the equilibrium was still not reached until 10^18^ arbitrary time units. Dotted vertical line indicates when the simulation reaches its equilibrium. The LMs that appear in both kinetics plots are marked with same color, otherwise pseudoknot-free and pseudoknotted LMs are marked with black and red, respectively. The sums of the structure probabilities of pseudoknot-free and pseudoknotted LMs on BHG${}^{\psi }$ are marked with blue and red dashed lines, respectively (Color version of this figure is available at *Bioinformatics* online.)

We compare the times for the molecule to reach its thermodynamic equilibrium in BHG${}^{\psi }$ and BHG${}^{{}^{\circ}}$, respectively. In most examples, the time to equilibrium is nearly the same. For example, the folding kinetics of the *Bacillus subtilis* transcriptional riboswitch preQ_1_ (36 nt) is shown in Figure 3 (left). In some cases, such as the *Escherichia* *coli* rRNA fragment (94 nt) in Figure 3 (middle), we find that pseudoknotted LMs significantly accelerate the folding kinetics. This can be explained by the appearances of some lower energy re-folding paths with pseudoknotted LMs. Finally, folding can be slowed down when pseudoknotted kinetic traps appear in the landscape, as in the signal recognition particle RNA (Fig. 3, right). Results for more RNAs are collected in SM Part G.

Since the BHG is built based on a sampling procedure, one might be concerned about the robustness of our results. We therefore repeated each analysis 10 times starting from independent sampling runs. As seen in Supplementary Table S5, saddle heights between low-energy LMs vary little between runs. Therefore, we conclude that folding dynamics derived based on these saddle heights are fairly stable.

### 3.2 A case study: SV11

The 115 nt SV11 RNA was discovered in *in vitro* selection experiments as an excellent substrate for Qβ replicase (Biebricher and Luce, 1992). It features a nearly palindromic sequence with an extremely stable, hairpin-like ground-state structure which does not contain any pseudoknot. Pulse-chase experiments showed that the active conformation is a metastable structure formed during replication, while the ground-state structure (with energy –95.9 kcal/mol) does not serve as a template for the Qβ replicase. Melting experiments indicated that the metastable structure (pseudoknot-free) with energy –63.6 kcal/mol comprises two distinct stems (Biebricher and Luce, 1992). The energy difference between the ground state and the metastable structure is 32.3 kcal/mol, well beyond the reach of exhaustive enumeration. In both BHG${}^{\psi }$ and BHG${}^{{}^{\circ}}$, the ground state is correctly predicted as the MFE structure and the metastable structure is detected as an LM ranked as 47 478 in BHG${}^{\psi }$ and 2466 in BHG${}^{{}^{\circ}}$ according to its free energy.

To further investigate the influence of pseudoknots on refolding between the metastable and the MFE structure, we constructed and compared optimal refolding paths in BHG${}^{\psi }$ and BHG${}^{{}^{\circ}}$. Even though we considered only paths with a peak energy equal to the saddle height and despite the coarse graining provided by the BHG, the number of paths connecting the two minima remains very large. Moreover, there is no common criterion to define which among these paths is the ‘best’. We therefore employed two alternative criteria to define the optimal folding path $U=(x_{0},x_{1}\ldots ,x_{k})$. (A) *U* minimizes the peak energy $\max_{s}\{f(x_{s})\}$ and, among equally good solutions, minimizes the accumulated activation energy, $\sum_{s}\left\{\text{S(}x_{s},x_{s+1})-f(x_{s})\right\}$. (B) *U* is a maximum likelihood trajectory with an upper time bound *T_m_* as introduced by Perkins (2009). Consider a trajectory $U=(x_{0},t_{0},x_{1},t_{1},\ldots ,x_{k-1},t_{k-1},x_{k})$ where the *x_i_* are the consecutive states and the *t_i_* are the waiting time in state *x_i_*, i.e. *x_i_* stays in state *x_i_* for a time *t_i_* and then transitions to state $x_{i+1}$ until time *T_m_.* The likelihood of *U* is

(2) $$L(U)=\prod_{i=0}^{k-1}\left(\lambda_{x_{i}}\cdot e^{-\lambda_{x_{i}}t_{i}}\cdot P_{x_{i},x_{i+1}}\right)\cdot e^{-\lambda_{x_{k}}(T_{m}-\sum t_{i})}$$

whenever $\sum t_{i}\leq T_{m}$, t and L(U)=0 otherwise. Since $\lambda_{x_{i}}=\sum_{x_{j}}r_{x_{i}x_{j}}$ and $P_{x_{i},x_{i+1}}=r_{x_{i+1},x_{i}}/\lambda_{x_{i}}$ in our model, Equation (2) simplifies to

(3) $$L(U)=\prod_{i=0}^{k-1}r_{x_{i}x_{i+1}}\cdot e^{-\left(\sum_{i=0}^{k-1}\lambda_{x_{i}}t_{i}+\lambda_{x_{k}}(T_{m}-\sum t_{i})\right)}.$$

**Fig. 4.**
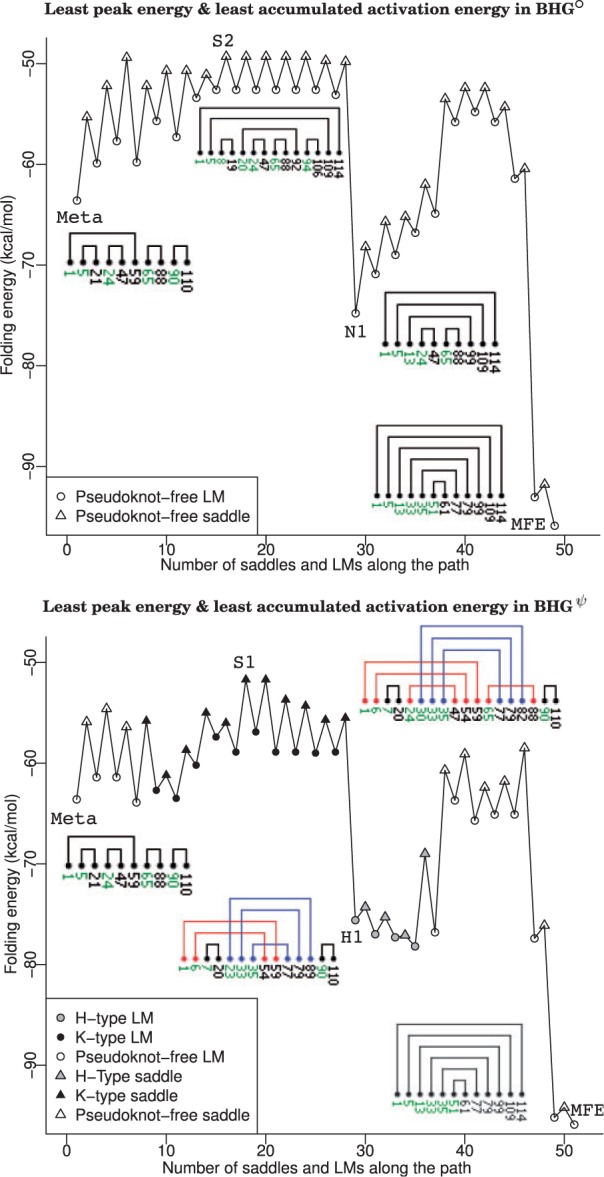
Folding energy profile of the optimized refolding paths of SV11 in BHG${}^{{}^{\circ}}$ (top) and BHG${}^{\psi }$ (bottom) from metastable state (Meta) to ground-state structure (MFE) according to Criterion A, i.e. the path with the least peak and accumulated energies. In which, only the LMs and saddles along the original refolding paths are shown in the folding energy profile for the sake of clarity. Helix representations of representative LMs and saddles are labeled along the refolding path. These helix representations are drawn by PseudoViewer (Han *et al.*, 2002). Only the outermost base pair in each helix of the structure is drawn with its terminating nucleotide indices annotated (Color version of this figure is available at *Bioinformatics* online.)

The optimal folding path according to Criterion A for BHG${}^{\psi }$ is shown in Figure 4 (bottom). This refolding path needs to pass through 26 LMs and has a saddle height of –51.7 kcal/mol. In contrast, the refolding path in BHG${}^{{}^{\circ}}$ shown Figure 4 (top) goes through 25 LMs with a slightly larger saddle height of –49.3 kcal/mol.

To illustrate the refolding paths more explicitly, we use the helix representations introduced by PseudoViewer (Han *et* *al.*, 2002). In this representation, only the outermost base pair in each helix of the structure is drawn with its terminated nucleotides indices annotated. For example, the metastable structure of SV11 includes five helices, denoted by $h_{1}^{59},\;h_{5}^{21}$, $h_{24}^{47},\;h_{65}^{88}$ and $h_{90}^{110}$. The subscripts and superscripts refer the start and end locations of the outermost base pair of the corresponding helix. The helix $h_{1}^{59}$ embraces two other helices $h_{5}^{21}$ and $h_{24}^{47}$ to form a multi-loop. In the BHG${}^{\psi }$, the refolding path visits 10 K-type and four H-type pseudoknotted LMs. After a *K*-type saddle structure S1 (–51.7 kcal/mol), the molecule forms two additional base pairs G28–C84 and C29–G83 in order to compensate the energy cost of decomposing $h_{65}^{88}$. Subsequently, the decomposition helices $h_{1}^{59}$ and $h_{6}^{54}$ in the H-type LM H1 leaves the rest of the path pseudoknot-free. The pseudoknot-free refolding path in BHG${}^{{}^{\circ}}$ made some local adjustments inside helix $h_{5}^{109}$ in order to compensate the energy cost of decomposing helices $h_{8}^{19}$ and $h_{94}^{106}$ in S2 to form an intermediate ‘valley point’ N1 which pushed the refolding fluctuation around its peak point.

For Criterion B, we consider two cases, with the upper time limit set to either *T* = 0 and $T=10^{11}$ given that the actual refolding time is around 10^10^. When *T* = 0, any *t_i_* that an RNA molecule stays in a particular state *s_i_* has to be 0 as well in order to maximize the likelihood. An interesting observation is that in this case Criterion B is equivalent to minimizing the accumulated activation energy. The optimal path in BHG${}^{\psi }$ and BHG${}^{{}^{\circ}}$ stays the same. It goes through a total of 19 LMs with log-likelihood –154.00, accumulated activation energy is 94.91 kcal/mol, and peak energy –38.6 kcal/mol. This optimal path does not go through any pseudoknotted LM. Comparing to the optimal paths according to Criterion A (with log-likelihood value –178.24 and accumulated activation energy 109.85 kcal/mol in BHG${}^{{}^{\circ}}$; –191.44 and 118 kcal/mol in BHG${}^{\psi }$), the molecule is inclined to have the overall shape of the MFE structure in a more ‘ambitious’ way rather than making detours through the landscape for lowering down the peak energy. When $T=10^{11}$, the optimal folding paths in BHG${}^{\psi }$ and BHG${}^{{}^{\circ}}$ are the same. It goes through 21 LMs with accumulated activation energy 109.85 kcal/mol and all of the LMs are pseudoknot-free. Further details can be found in SM Part H.

**Fig. 5.**
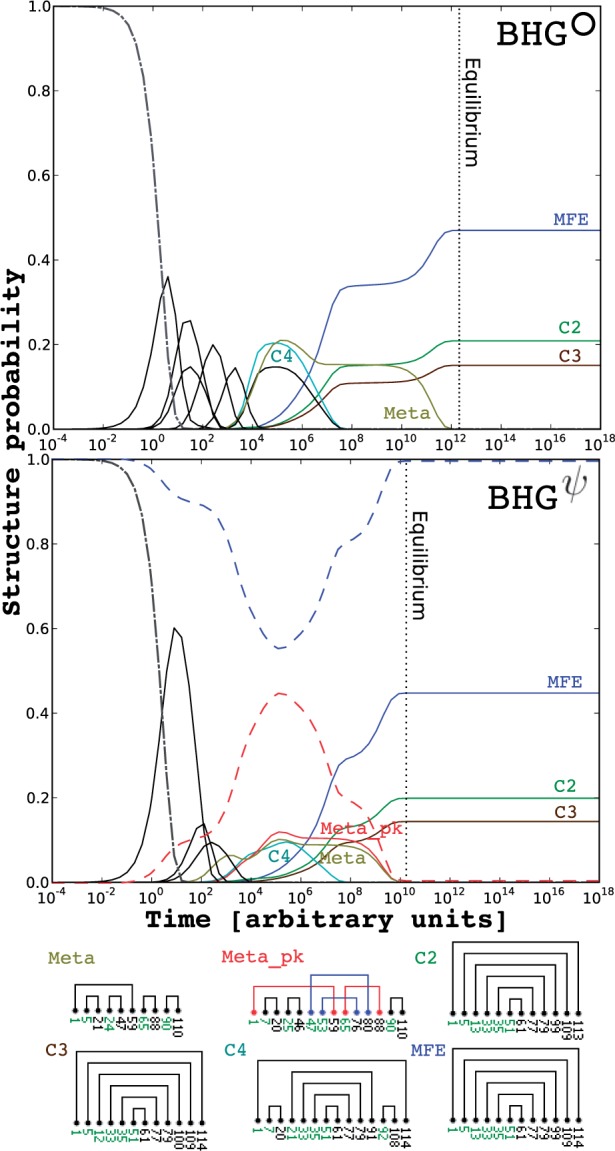
Folding kinetics of SV11 RNA switch L07337_1 based on BHG${}^{{}^{\circ}}$ (top) and BHG${}^{\psi }$ (bottom). Both simulations were started in the open chain structure and run until convergence to the thermodynamic equilibrium distribution. Only LMs whose population probabilities reach 14.5% (BHG${}^{{}^{\circ}}$) and 9% (BHG${}^{\psi }$) are visualized for the sake of clarity. The state space of this simulation is reduced to 10^4^ LMs from 175 733 LMs on BHG${}^{\psi }$ (BHG${}^{{}^{\circ}}$ contains 8105 LMs, so no further reduction is applied). The *x*- and *y*-axes indicate the time and population probabilities, respectively. Dotted vertical line indicates when the simulation reaches its equilibrium. The LMs that appear in both kinetics plots are marked with same color, otherwise black. The only exceptional case is the Meta_pk, which is marked red for highlighting purposes. The sums of the population probabilities of pseudoknot-free and pseudoknotted LMs on BHG${}^{\psi }$ are marked with blue and red broken lines, respectively (Color version of this figure is available at *Bioinformatics* online.)

Finally, we compare the BHG${}^{\psi }$-based folding kinetics simulation to the simulation based on BHG${}^{{}^{\circ}}$. As shown in Figure 5, the BHG${}^{\psi }$-based simulation reached its equilibrium earlier than the BHG${}^{{}^{\circ}}$ case. The metastable state is populated from around $t=10^{3}$ to 10^12^ in the BHG${}^{{}^{\circ}}$-based simulation and from *t* = 10 to 10^10^ in the BHG${}^{\psi }$ case. Our simulation based on BHG${}^{\psi }$ suggested that there exists another long-lived metastable structure Meta_pk with a *K*-type pseudoknot. Meta_pk has energy –64.0 kcal/mol and has nearly the same life time as Meta (–63.6 kcal/mol). In particular, the period of time during which the MFE structure gains population from the decay of Meta is nearly the same as the case of Meta_pk. This is because Meta_pk and Meta are separated by the same energy barrier from the MFE structure.

## 4 Concluding remarks

We have demonstrated here that it is computationally feasible to compute BHGs for secondary structures with a broad class of pseudoknots. The basin hopping graphs BHG${}^{\psi }$ and BHG${}^{{}^{\circ}}$ are comparable for RNAs with pseudoknot-free ground states. Therefore, they can be used to investigate the effects of pseudoknots in the folding process. We observe that for the majority of such RNAs the inclusion of pseudoknots makes little difference for the time to reach equilibrium. However, there are RNAs where pseudoknots substantially speed up the folding process by lowering the energy barrier. On the other hand, pseudoknotted structures may also appear as kinetic trap states and prevent efficient folding.

Our observations suggest that pseudoknotted structures *should* be included in the analysis even when main states of an RNA switch are pseudoknot free. Pseudoknots do, however, incur significant computational cost, both because pseudoknot prediction methods are expensive ($O(n^{6})$ in case of gfold) and even more so, because the size of landscape grows. Pseudoknotted folding intermediates that lower the energy barrier are almost always H-type or K-type. This suggests that the more complex L-type and M-type pseudoknots could be neglected, while the ability to predict kissing hairpins is essential for a realistic description of RNA folding landscapes. Unfortunately, most current methods for pseudoknot prediction focus solely on the prediction of ground-state structures, while our approach requires the ability to sample structures from the Boltzmann ensemble. Apart from this requirement, any other method could be used as a drop-in replacement for gfold.

## Supplementary Material

Supplementary Data
