# Supplementary material for: Pseudoknots in RNA folding landscapes
Source: Bioinformatics. 2015 Oct 1;32(2):187–94. doi: 10.1093/bioinformatics/btv572 (PMC4708108; doi:10.1093/bioinformatics/btv572)
Supplement: Supplementary Data [file supp_32_2_187__index.html]

Pseudoknots in RNA folding landscapes — Pseudoknots in RNA folding landscapes — Supplementary Data 

# Pseudoknots in RNA folding landscapes

## Supplementary Data

files

- Supplementary Data - pdf file
