# Supplementary material for: Pseudoknots in RNA folding landscapes
Source: Bioinformatics. 2015 Oct 1;32(2):187–94. doi: 10.1093/bioinformatics/btv572 (PMC4708108; doi:10.1093/bioinformatics/btv572)
Supplement: Supplementary Data [file supp_btv572_supp1.pdf]

BIOINFORMATICS
Vol. 00 no. 00
Pages 1–19

Pseudoknots in RNA folding landscapes SUPPLEMENTAL MATERIAL

Marcel Kucharík[1], Ivo L. Hofacker[1–3], Peter F. Stadler[1,4–7], and Jing Qin[1,3,8]

[1]Institute for Theoretical Chemistry, Univ. Vienna, Währingerstr. 17, 1090 Vienna, Austria
[2]Research group BCB, Faculty of Computer Science, Univ. Vienna, Austria
[3]RTH, University of Copenhagen, Grønnegårdsvej 3, Frederiksberg, Denmark
[4]Dept. of Computer Science & IZBI & iDiv & LIFE, Leipzig Univ., Härtelstr. 16-18, Leipzig, Germany
[5]Max Planck Institute for Mathematics in the Sciences, Inselstr. 22, Leipzig, Germany
[6]Fraunhofer Institute IZI, Perlickstr. 1, Leipzig, Germany
[7]Santa Fe Institute, 1399 Hyde Park Rd., Santa Fe, NM87501, USA
[8]IMADA, Univ. Southern Denmark, Campusvej 55, Odense, Denmark.

1 PART A: RNA FOLDING LANDSCAPES AND BASIN HOPPING GRAPH REVISITED

Given an RNA sequence σ, in this contribution, we consider the ensemble $X = X_\sigma$ of secondary structures in which pseudoknots can be included. It has been proven that the cardinality $|X_\sigma|$ grows exponentially with the length of σ (Akutsu (2000); Lyngso & Pedersen (2000) and the references therein) provided the stickiness of σ, i.e., the probability that two arbitrarily chosen nucleotides in σ can form a base pair, is relatively large. This is true for most biological RNA sequences, since the values of stickiness for RNAs are around 0.375 (Hofacker *et al.*, 1994).

This ensemble of RNA structures can be arranged as a graph, referred as RNA folding landscape, by defining a "move set", i.e. by specifying which pairs of secondary structures can be interconverted in a single step (Reidys & Stadler (2002) and the references therein). Each vertex of the RNA folding landscape, i.e., each RNA secondary structure x, is associated with an energy $f(x)$. For the cases of pseudoknot-free structures, a well-established energy model allows us to explicitly compute $f(x)$ for every structure s in terms of additive contributions for base pair stacking as well as hairpin loops, interior loops, bulges, and multiloops (Mathews *et al.*, 1999). When pseudoknots appear, the evaluation of free energy gets more involved. The current energy models for pseudoknots are simple, heuristic extensions of the standard energy model that use "developer-defined" energy penalties to score pseudoknots. An alternative, rather general energy function for pseudoknotted structures has been derived from the "cross-linked gel model" (Isambert & Siggia, 2000), however it suffers from the same lack of experimental data. Furthermore, no open source implementation of this energy function is available.

A structure $x \in X$ is a *local minimum* (LM) of the landscape if it does not have neighbors with lower energy. In particular, x is a *global minimum* or a *minimum free energy* structure (MFE) if its energy is minimal within X. For each LM x we define its *gradient basin* $G(x) \subset X$ as the set of structures $z \in \mathsf{X}$ so that the unique gradient walk with starting point in z ends in x. We note for later reference that the gradient basins of all the LMs in the RNA folding landscape forms a *partition* of its configuration space X. This partitioning forms an intuitive coarse-grained model of the landscape.

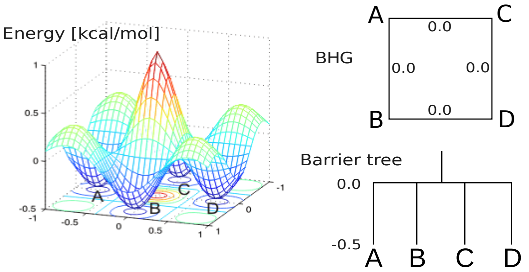

Fig. 1. A landscape with four local minima (A, B, C, and D) is illustrated in the left hand side. Its corresponding barrier tree (bottom) and basin hopping graph (top) are shown on the right hand side with saddle heights annotated inside. For any pair of local minima, their corresponding saddle heights are all equal to 0 kcal/mol. Regarding direct saddle heights, expect $\mathsf{DS}(A, D) = \mathsf{DS}(B, C) = 0.5$kcal/mol, the remaining are all of value 0 kcal/mol. One key difference is the energetically favorable neighborhood relation between the basins, can be displayed in the basin hopping graph, but *not* in the barrier tree.

The *cycle* $B_h(x)$ of x at energy level h can be defined as a maximal connected subset of $\{z \in X | f(z) \leq h\}$ that contains x. In other worlds, $B_h(x)$ is the set of structures found by a flooding algorithm starting at x (Sibani *et al.*, 1999; Flamm *et al.*, 2000, 2002). In particular, the basin $B(s) = B_{f(s)}(s)$ of s (Flamm *et al.*, 2002) is the set of all points in X that can be reached from s by a path whose elevation never exceeds $f(s)$.

A *direct saddle* between two LMs x and y is a structure $s \in X$ with minimal energy so that both x and y are reachable from s by means of an adaptive walk. We call $\mathsf{DS}(x, y) = f(s)$ the direct saddle height between x and y. Not every pair of LMs is connected by direct saddles.

The *saddle height* $\mathsf{S}(x, y)$ between any two vertices x and y is the minimal value h for which $y \in B_h(x)$. In other words, $\mathsf{S}(x, y)$ is the level at which two cycles $B_h(x)$ and $B_h(y)$ "merge". If x and y are LMs connected by a direct saddle point then $\mathsf{S}(x, y) \leq \mathsf{DS}(x, y)$. A structure $s \in \mathsf{X}$ is called a *saddle* between $x, y \in X$ if (i) $f(s) = \mathsf{S}(x, y)$ and (ii) there is a path P connecting x and y so that $f(s) \geq f(z)$ for all $z \in P$. A path P^* connecting x and y in the landscape is *energetically optimal* if $\max_{z \in P^*} f(z) =$

© Oxford University Press 2015.

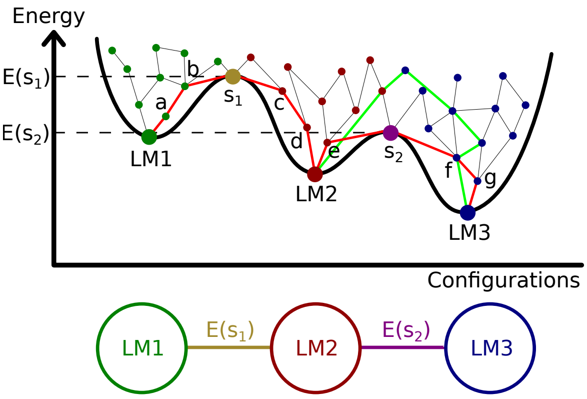

Fig. 2. Saddles, direct saddles and energetically optimal paths. (Top) The y-axis denotes the (free) energies of the structures in the landscape. There are in total three gradient basins with local minima LM1, LM2 and LM3. Structures in the same gradient basin are labeled with the same color, except two saddles s_1 and s_2. In which, the structure s_1 is a direct saddle and saddle between LM1 and LM2. An energetically optimal path between LM1 and LM2 pass through structures a, b, s_1, c, d consecutively. Analogously, s_2 is a direct saddle and saddle between LM2 and LM3 with an energetically optimal path passing through structures e, s_2, f, and g. Note here, s_1 is a saddle but *not* a direct saddle between LM1 and LM3. In fact, there does not exist any direct saddle between LM1 and LM3 since there does not exist any structure from which both LM1 and LM3 are reachable by adaptive walks. (Bottom) The resulting BHG of this landscape.

$\mathsf{S}(x, y)$. Energetically optimal paths are not necessarily unique. See Fig. 2 for an illustration of the concepts mentioned above. For RNA folding landscapes, the problems of computing saddle heights, saddle points and energetically optimal path are NP-hard (Maňuch *et al.*, 2011).

The basic idea of basin hopping graph (BHG) is to incorporate additional neighborhood information by considering LMs as adjacent if the transition between their corresponding basins are “energetically optimal”. A schematic diagram of BHG for a toy landscape is illustrated in Fig. 1. In which, the transition from A to B on Fig. 1 is energetically optimal, since $\mathsf{S}(A, B) = \mathsf{DS}(A, B) = 0$, but the transition from A to D is not, since $1 = \mathsf{DS}(A, D) > \mathsf{S}(A, D) = 0$.

2 PART B: IMPLEMENTATION DETAILS OF PSEUDOKNOTS

2.1 Pseudoknot energy model of 1-structures in `gfold`

In this section, we give a brief review of the energy model for evaluating 1-structures introduced in `gfold` (Reidys *et al.*, 2011). A full-fledged version is available in the supplementary material of their original paper.

In the pseudoknot energy model of `gfold`, except pseudoknotted loops, all other types of loops are evaluated according to the standard Turner 1999 energy model (Mathews *et al.*, 1999). The energy contributions of pseudoknotted loops are evaluated as an extended version of multiloops.

More precisely, the energy of an external pseudoknot (a pseudoknot not covered by any base pair) is evaluated as

$$E^{pseudo} = \beta_{Type} + B \cdot \beta_2 + U \cdot \beta_3. \tag{1}$$

In which, the parameter β_{Type} is is the penalty of forming a pseudoknot of Type H, K, L, or M, B is the number of base pairs forming the pseudoknot, and U is number of unpaired nucleotides inside the loop.

Since the number of crossing base pairs is always at least two, a multiloop is formed whenever a pseudoknot is nested in a base pair. In these cases, the penalty parameter β_{Type} is replaced by β_{Type}^{mul}. Otherwise, if a pseudoknot is nested in another pseudoknot, then β_{Type} is replaced by β_{Type}^{pseudo}. The energy parameters for pseudoknots used in the `gfold` are listed in Table 1.

There is a heavy penalty for forming a pseudoknot inside another pseudoknot or multiloop, which may be due to a lack of experimental evidence of such complicate pseudoknotted structures. As a result of the relatively heavy penalties to form a pseudoknot, a gradient walk starting from a pseudoknot-free structure can not end in a pseudoknotted LM.

Table 1. The energy parameters for pseudoknot used in `gfold`. All energy values are evaluated in units of kcal/mol.

Type=	H	K	L	M
β_{Type}	9.6	12.6	14.6	17.6
β_{Type}^{mul}	15.0	18.0	20.0	23.0
β_{Type}^{pseudo}	15.0	18.0	20.0	23.0
β_2	0.1			
β_3	0.1			

2.2 Adaptations in `gfold` program

We have made some necessary adaptions in `gfold` (Bon *et al.*, 2008; Reidys *et al.*, 2011) to implement the adaptive sampling schedule used in `RNAlocmin`. First, an additional option for the ξ-scaling procedure required in `RNAlocmin` is implemented. Secondly, the output format of `gfold` is tailored for its usages in the ξ-scaling procedure including an option to vary the sample sizes. The original output file of `gfold` is still kept as an output option in the modified version, which is available on the webpage `https://github.com/marcelTBI/gfold`.

2.3 Energy parameters in BHG$^\psi$ and BHG$^\circ$

For comparison purpose, in this publication we often consider, for an RNA whose ground state is pseudoknot-free the full BHG$^\psi$ including pseudoknotted LMs and a pruned BHG$^\circ$ from which first all pseudoknotted LMs are removed and then the BHG-adjacency is recomputed using only pseudoknot-free structures along the paths.

To make sure that the energy parameters are coherent, in both BHG$^\psi$ and BHG$^\circ$, we are obliged to use the standard Turner energy model (Mathews *et al.*, 1999) without considering dangle energies as implemented in the ViennaRNA Package with options
`-d0 -P rna_turner99.par`.

This is because in the energy model used in `gfold`, the penalty-parameters for pseudoknots are *only* trained under the standard Turner energy model (Mathews *et al.*, 1999) at $37^\circ C$ without taking the dangle energies into consideration.

2.4 Determine valid base pairs to add into a secondary structure

Given a 1-structure S, we first need to construct the *conflict graph* of S. The vertices of *conflict graph* are constructed based on the relations between any two helices $a = (l_a, r_a; d_a)$ which is a set of base pairs $\{(l_a, r_a), (l_a + 1, r_a - 1), \dots, (l_a + d_a, r_a - d_a)\}$ and $b = (l_b, r_b; d_b)$ of S:

1. crossing, denoted by $a \perp b$ if $l_a < l_b < r_a < r_b$ or its symmetric case is true;
2. nesting, denoted by $a \| b$ if $l_a < l_b < r_b < r_a$ or its symmetric case is true;

These two relations give rise to a partition of the helices of S into *gap-sets* via requiring that two helices a and b belong to the same gap-set if $a \| b$ and they cross with the same set of helices in S. For example, in Fig. 3 (A), there are in total 6 helices $\{a_1, a_2, \dots, a_6\}$ in a pseudoknotted structure and 5 gap-sets $\{\{a_1, a_6\}, \{a_2\}, \{a_3\}, \{a_4\}, \{a_5\}\}$. Each gap-set is represented as a vertex in its conflict graph shown in Fig. 3 (B). Furthermore, we draw an edge in the conflict graph between two vertices, if their corresponding helices cross with each other. In Fig. 3 (B), two gap-sets $\{a_4\}$ and $\{a_1, a_6\}$ are adjacent in the conflict graph given that $a_1 \perp a_4$ and $a_6 \perp a_4$.

Adding a base pair a in S therefore, in the "worst" case, is equivalent to add a vertex (and potential incident edges) into the conflict-graph of S accordingly, see Fig. 3 (D) for an example. Thus all we need is to test whether the components of the resulting conflict graph has some component other than the 5 valid types shown in Fig. 3 (C). In particular, we only need to consider the components which contain base pairs crossing with a.

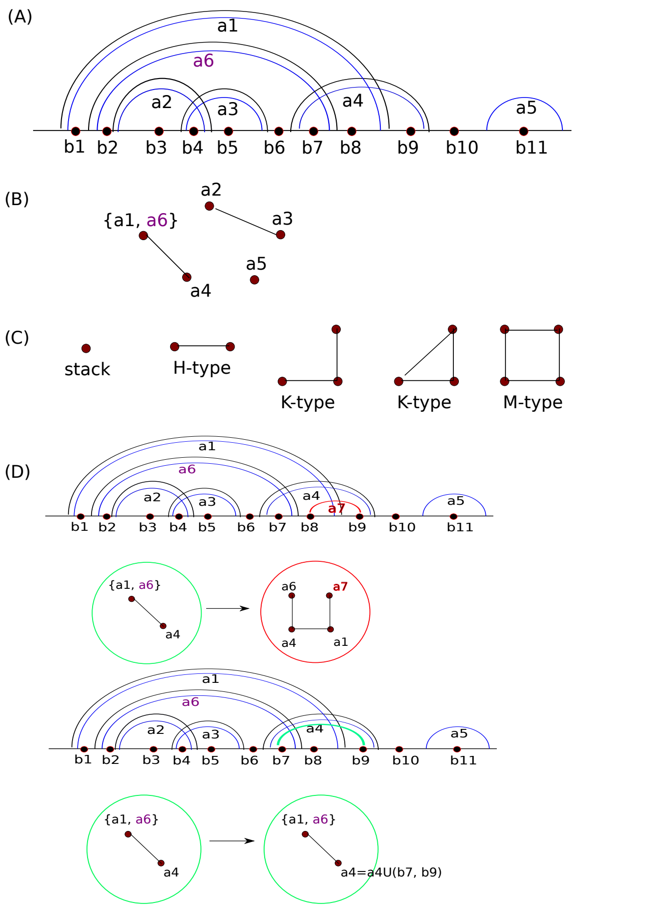

Fig. 3. Given a pseudoknotted structure (A) and its conflict-graph (B). This structure has in total 6 helices notated with $\{a_1, a_2, \dots, a_6\}$ and 11 single-stranded nucleotides notated with $\{b_1, b_2, \dots, b_{11}\}$. Six helices are divided into 5 gap-sets gap-sets $\{\{a_1, a_6\}, \{a_2\}, \{a_3\}, \{a_4\}, \{a_5\}\}$ which accordingly become the vertices in its conflict graph. Adding a new base pair $a_7 = (b_8, b_9)$ gives rise to an invalid structure since the component including a_7 in the resulting conflict graph is not one of the five valid types shown in (C). Adding a base pair (b_7, b_9) gives rise to a valid structure since the according conflict graph stays the same except the helix a_4 thickens by the base pair (b_7, b_9).

3 PART C: DETAILS OF THE RNA FOLDING KINETICS

3.1 Methods

From a microscopic point of view, the dynamics on an RNA folding landscape can be described by a continuous-time Markov process with infinitesimal generator $\mathbf{R} = (r_{yx})$ (Flamm *et al.*, 2000). The transition rate r_{yx} from a secondary structure x to y is non-zero only if x and y are adjacent, i.e., if they differ by adding/removing a single base pair. Typically the Metropolis rule, the following formula is used to assign microscopic rates

$$r_{yx} = r_0 \min\{\exp\{-(f(y) - f(x))/RT\}, 1\}. \quad (1)$$

Here, f evaluates the (free) energy of x, R is the universal gas constant, T is the absolute ambient temperature and r_0 is a parameter used to gauge the time axis from experimental data. Here we simply use $r_0 = 1$, implicitly defining our time unit.

Denote the probability that an RNA molecule has the secondary structure x at time t by $P_{x,t}$, the dynamics is governed by the master equation $dP_{x,t}/dt = \sum_y r_{xy} P_{y,t}$ with $r_{xx} = -\sum_{y \neq x} r_{yx}$. This linear system of differential equations can be exactly solved by explicitly computing $\mathbf{P}(t) = \exp(t\mathbf{R}) \cdot \mathbf{P}(0)$ for short RNA molecules $\sim$ 30nt, where $\mathbf{P}(t)$ is the vector of $P_{x,t}$ for all possible structures x. The program `treekin` (Wolfinger *et al.*, 2004) provides an implementation of this method.

Even for RNA molecules of moderate size, direct computation of the matrix exponential becomes impossible due to the exponential growth of the underlying state space. An alternative is to perform stochastic simulations as is done in the `kinfold` program Flamm *et al.* (2000), however this turns out to be rather time consuming for large RNA molecules. Wolfinger *et al.* (2004) used barrier trees (Flamm *et al.*, 2002) to assign a macro state to each local minimum and recalculate rates between these. This approximation has shown excellent agreement to the full-process computed from Eqn. 1 with all possible structures, but its exhaustive nature limits its applicability to molecules up to $\sim$ 80nt.

We observed that the computation of matrix exponentials in `treekin` becomes numerically unstable when some transition rates are very small. We therefore use a Padé approximation and the scaling and squaring method described in (Al-Mohy & Higham, 2009) and implemented in the function `f01ecc` of the NAG library Mark 9 with time complexity of $O(N^3)$ (N is the dimension of the matrix and thus the number of the LMs in our case).

3.2 Comparison to Wolfinger *et al.* (2004)'s folding dynamic approximation

To demonstrate the quality of the BHG approximation, we present the comparison to the barrier tree based coarse graining of folding kinetics for several examples. We show that our approximation reflects a qualitatively correct description of the process, as well as important quantitative details, such as, the ordering of the top frequent structures and the time needed to converge to the thermodynamic equilibrium distribution. The time for an RNA to reach the equilibrium is evaluated as the first time t, when the Euclidean distance between computed distribution $\mathbf{P}(t)$ and the Boltzmann equilibrium distributions is less than a threshold 10^{-5}.

The examples include the following: the *Pyaiella Littoralis* Group II Intron (PDB_01042, 34nt, Fig. 4), the pseudoknot domain of tmRNA from E. coli (PKB49, 30nt, Fig. 5) and Legionella pneumophila (PKB67, 30nt, Fig. 6), a synthetic tetraloop-receptor (PDB_00924, 86nt, Fig. 7), and a Hammerhead ribozyme (type III) (RFA_00398, 54nt, Fig. 8). The LMs that appear in both kinetics plots are marked with same color, otherwise with black.

For longer RNAs, the exponential growth of LMs in the BHG poses computational difficulties in our continuous time Markov chain based folding simulations, since the number of LMs considered is exactly the dimension of the infinitesimal generator $\mathbf{R}$. The number of LMs on BHG can be easily beyond 10^5 for an RNA of length $\sim$ 100nt, even with additional restriction on their energy range. Furthermore, our observations show that only a small portion of the whole set of LMs on BHG play important roles in the kinetic simulations, most of the LMs only contribute in fast fluctuations and the resulting computational cost. For example, the folding kinetics of the *Pyaiella Littoralis* Group II Intron shown in Fig. 4 is constructed from 185 LMs with Wolfinger *et al.* (2004)'s approximation and 173 LMs on BHG, respectively. But in both simulations, there are only 6 LMs whose population probabilities rises beyond 7% at any time during the kinetic simulations.

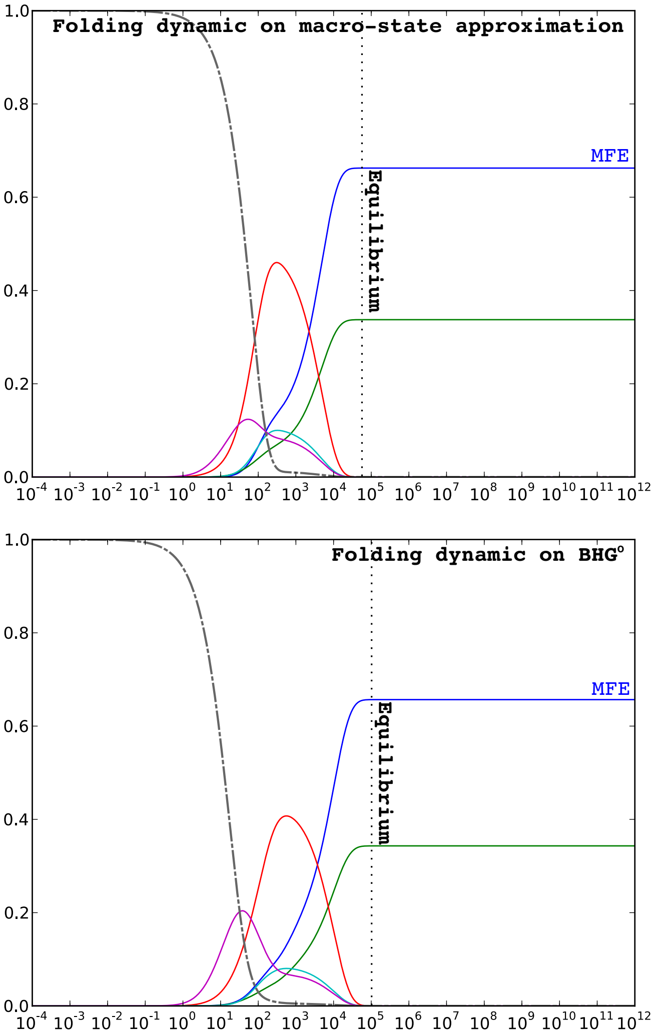

Fig. 4. Folding kinetics of the *Pyaiella Littoralis* Group II Intron (PDB_01042, 34nt). (Top) Wolfinger *et al.* (2004)'s folding dynamic approximation and (Bottom) Arrhenius approximation on BHG. The process was started in the open chain state and run until convergence to the thermodynamic equilibrium distribution. The x-axes and y-axes indicate the time and population probabilities, respectively.

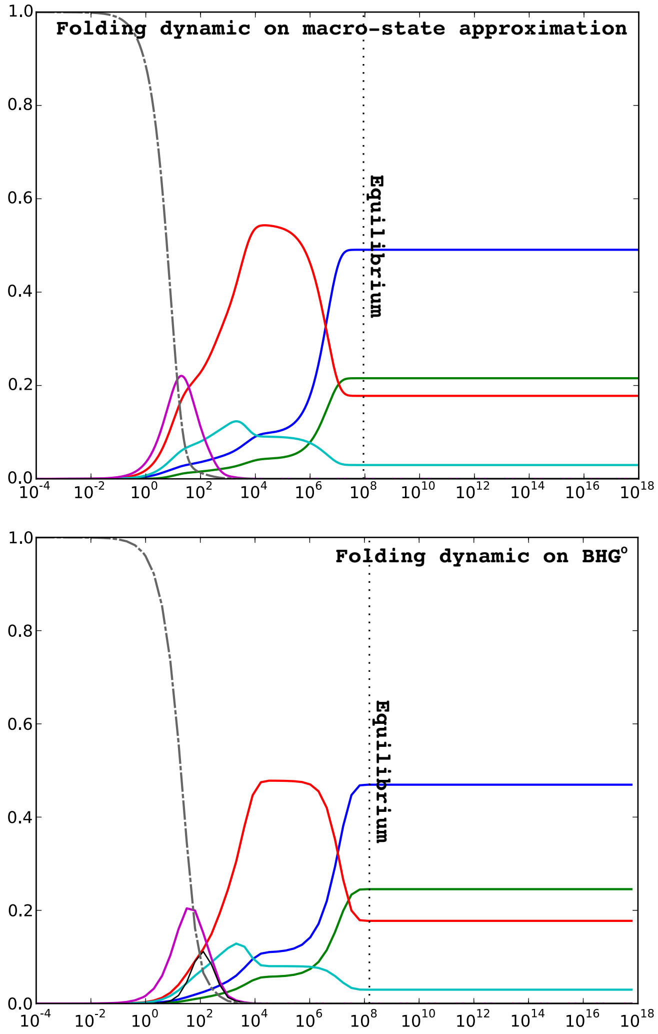

Fig. 5. Folding kinetics of the pseudoknot domain of tmRNA from E. coli (PKB49, 30nt). (Top) Wolfinger *et al.* (2004)'s folding dynamic approximation and (Bottom) Arrhenius approximation on BHG. The process was started in the open chain state and run until convergence to the thermodynamic equilibrium distribution. The x-axes and y-axes indicate the time and population probabilities, respectively.

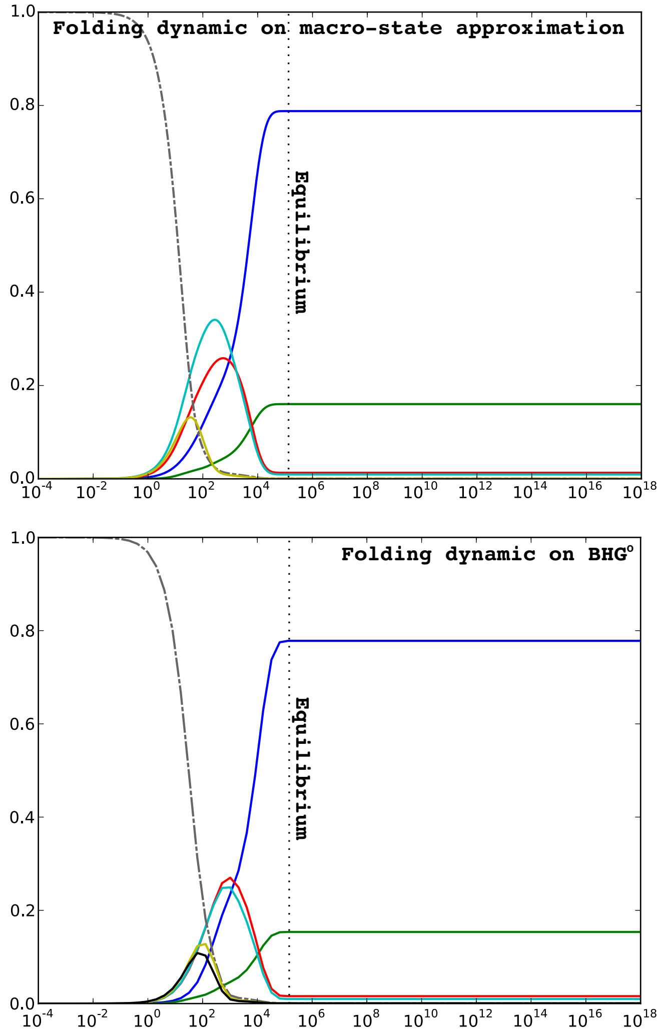

Fig. 6. Folding kinetics of the pseudoknot domain of tmRNA from Legionella pneumophila (PKB67, 30nt). (Top) Wolfinger *et al.* (2004)'s folding dynamic approximation and (Bottom) Arrhenius approximation on BHG. The process was started in the open chain state and run until convergence to the thermodynamic equilibrium distribution. The x-axes and y-axes indicate the time and population probabilities, respectively.

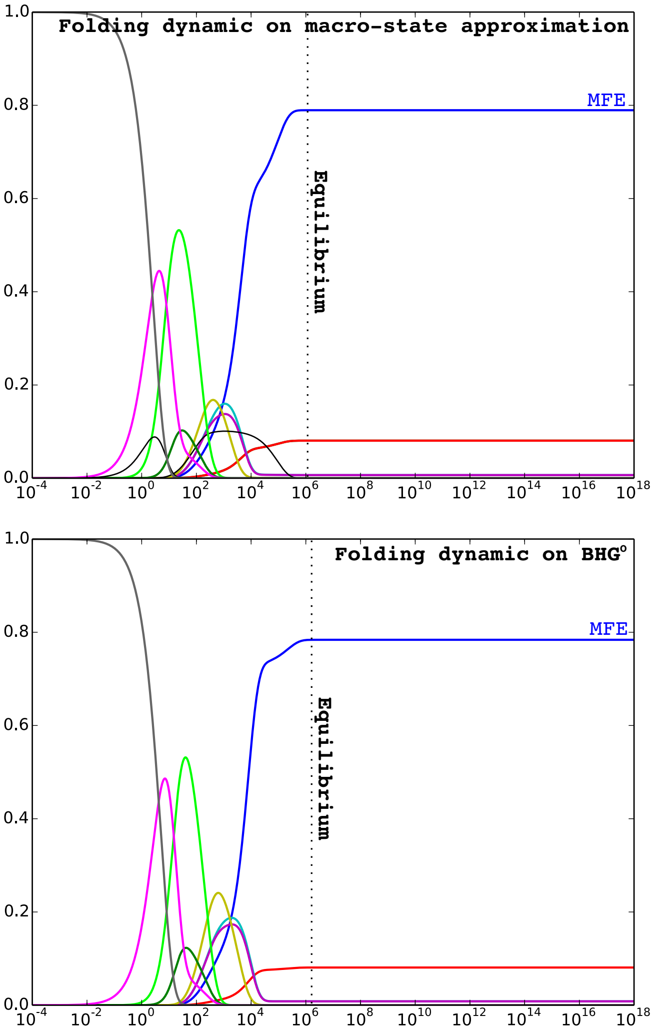

Fig. 7. Folding kinetics of a synthetic tetraloop-receptor (PDB_00924, 86nt). (Top) Wolfinger *et al.* (2004)'s folding dynamic approximation and (Bottom) Arrhenius approximation on BHG. The process was started in the structure
.......((((((((((((....))))))))))))((.(((....))).))((((((((..(....)..))))))))......... which is an LM of energy -37.80 kcal/mol. This kinetic process was not started in the open chain structure given that there are more than 10000 LMs in between the open structure and the ground state structure which is beyond the feasible range of the Wolfinger *et al.* (2004)'s folding dynamic approximation. The process was run until convergence to the thermodynamic equilibrium distribution. The x-axes and y-axes indicate the time and population probabilities, respectively.

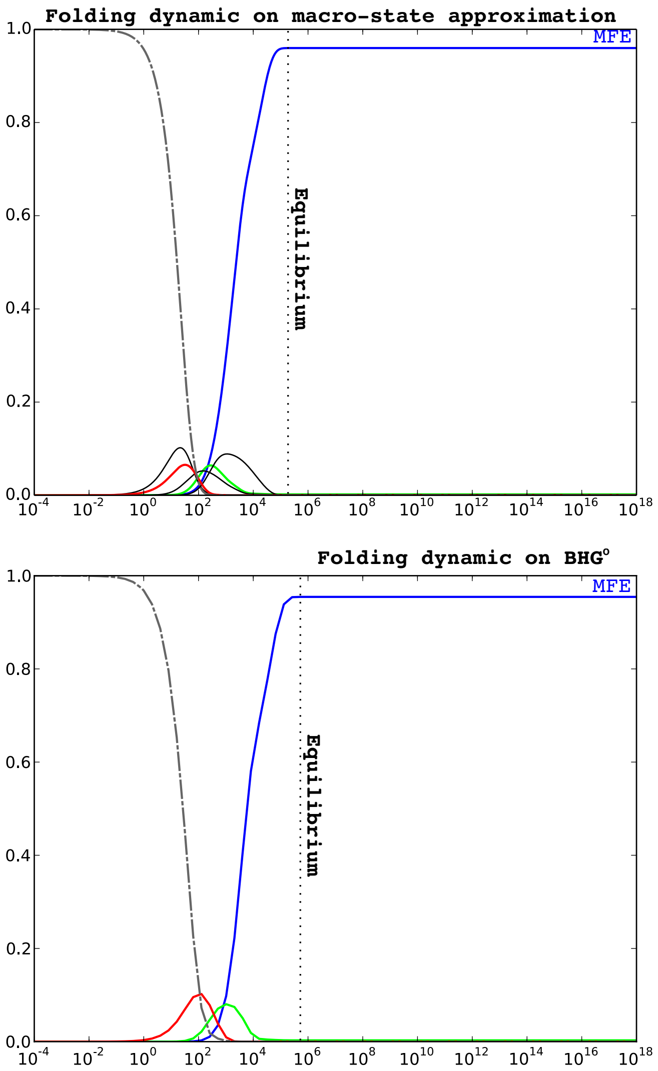

Fig. 8. Folding kinetics of the Hammerhead ribozyme (type III) (RFA_00398, 54nt). (Top) Wolfinger *et al.* (2004)'s folding dynamic approximation and (Bottom) Arrhenius approximation on BHG. The process was started in the open chain state and run until convergence to the thermodynamic equilibrium distribution. The x-axes and y-axes indicate the time and population probabilities, respectively.

4 PART D: QUASI-STEADY-STATE REDUCTION

We first partitioned all the LMs found into two categories: (G) important LMs (with high degree in our case) which are the "good" ones to keep and (B) intermediate LMs which are the "bad" ones to be neglected. Next, we re-arrange the ordering of the LMs based on their categories so that the rate matrix $\mathbf{R}$ and population vector $\mathbf{P}(t)$ can be rewritten into the following format

$$\begin{aligned}\mathbf{R} &= \begin{pmatrix}\mathbf{GG} & \mathbf{GB}\\ \mathbf{BG} & \mathbf{BB}\end{pmatrix}\\ \mathbf{P}(t) &= (\mathbf{P}_G(t), \mathbf{P}_B(t))\end{aligned}$$

In which, $\mathbf{P}_G(t)$ and $\mathbf{P}_B(t)$ denotes the population subvectors of the good and bad states respectively. Submatrix $\mathbf{GB}$ contains the transition rates from good states to bad states. The remaining three sub-matrices $\mathbf{GG}$, $\mathbf{BG}$ and $\mathbf{BB}$ are defined analogously.

Accordingly, $\frac{d\mathbf{P}(t)}{dt} = \mathbf{P}(t)\mathbf{R}$ can be written as

$$\left(\frac{d\mathbf{P}_G(t)}{dt}, \frac{d\mathbf{P}_B(t)}{dt}\right) = (\mathbf{P}_G(t), \mathbf{P}_B(t)) \cdot \begin{pmatrix}\mathbf{GG} & \mathbf{GB}\\ \mathbf{BG} & \mathbf{BB}\end{pmatrix}$$

Equivalently, we have

$$\begin{aligned}\frac{d\mathbf{P}_G(t)}{dt} &= \mathbf{P}_G(t)\cdot\mathbf{GG} + \mathbf{P}_B(t)\cdot\mathbf{BG}\\ \frac{d\mathbf{P}_B(t)}{dt} &= \mathbf{P}_G(t)\cdot\mathbf{GB} + \mathbf{P}_B(t)\cdot\mathbf{BB}\end{aligned}$$

Using $\frac{d\mathbf{P}_B(t)}{dt} = 0$, we derive

$$\mathbf{P}_B(t) = -\mathbf{P}_G(t)\cdot\mathbf{GB}\cdot\mathbf{BB}^{-1}$$

and furthermore

$$\mathbf{P}_G(t) = \mathbf{P}_G(0)\cdot e^{\mathbf{GG}-\mathbf{GB}\cdot\mathbf{BB}^{-1}\cdot\mathbf{BG}}.$$

In which, the Schur complement $\mathbf{GG} - \mathbf{GB}\cdot\mathbf{BB}^{-1}\cdot\mathbf{BG}$ can be computed efficiently given that the rate matrix $\mathbf{R}$ is sparse. Due to properties of Schur complement, it can be computed iteratively – reducing a single LM at each step (the matrix $\mathbf{BB}$ is a scalar). Then the time complexity of such a single step is $O(c^2)$, where c is the number of neighbors of this LM. Assign $b = dim(\mathbf{BB})$ and assume that degree of all reduced LM is small and bounded by some c_{max} ($c_{max} \ll b$), then the whole time complexity is $O(bc_{max}^2)$. However, if the matrix is dense ($c_{max} \sim b$) this reduction is equally time consuming as naive computation of $\mathbf{BB}^{-1}$ and thus unfeasible for our purposes.

In practice, this heuristic works reasonably well and has been implemented as part of the `BHGbuilder` program.

5 PART E: ANALYSIS OF THE LOWER PART OF RNA MOLECULES' LANDSCAPES

5.1 Summary of LMs and `gfold`-sampling structures in the lower parts of RNA molecules' landscapes

We analyze the composition of the LMs in the "lower" part of the energy landscapes of various RNA molecules listed in Table 2. In which, "lower" part means that we only consider LMs with negative free energies and within 10 kcal/mol above the minimum free energy of the whole folding landscape.

Table 2. Basic information of the RNAs including length (LEN) and type (TYPE)

ID	LEN	TYPE
PKB259	57	Viral 3 UTR
PKB139	67	Viral tRNA-like
PKB173	73	Ribozymes
PKB238	84	Viral 3 UTR
PKB138	116	Viral tRNA-like
PKB2	50	Viral ribosomal frameshifting
PKB49	30	tmRNA
PKB52	52	tmRNA
PKB67	30	tmRNA
PKB70	55	tmRNA
PKB71	108	mRNA
PDB_00213	101	Synthetic RNA
PDB_00542	126	Synthetic RNA
PDB_00702	94	Other Ribosomal RNA
PDB_00924	86	Synthetic RNA
PDB_01042	34	Group II Intron
RFA_00398	54	Hammerhead Ribozyme
SRP_00005	90	Signal Recognition Particle RNA
SRP_00094	91	Signal Recognition Particle RNA
SRP_00194	81	Signal Recognition Particle RNA
SRP_00284	87	Signal Recognition Particle RNA
TMR_00272	102	tmRNA
Bsu	42	Synthetic RNA

We contrast RNAs with pseudoknots in their ground state structures selected from `Pseudobase++` (PKB ID), (Han *et al.*, 2002; Taufer *et al.*, 2009) and pseudoknot-free structures from the `RNA STRAND` database (Andronescu *et al.*, 2008) (RNA STRAND ID). As well the transcriptional *Bacillus subtilis* riboswitch (Bsu) with an H-type pseudoknotted structure or a pseudoknot-free structure as its ground state depending on the presence of $preQ_1$ (Suddala *et al.*, 2013). Note here, we select the molecules such that their ground state structures predicted by `gfold` have both sensitivity and PPV beyond 80%, so that the effects caused by the prediction software can be limited.

The mean and standard deviation (STDEV) for the number and proportion of LMs of each type (pseudoknotted (H, K, L) or pseudoknot-free (N)) are given in Table 3 based on 10 independent samples. Furthermore, in Table 4 we report the analogous information of the `gfold`-sampling structures starting from which these LMs are derived by simulating gradient walks. In both tables, the numbers regarding to the M-type LMs or structures are omitted, since structures of such type were not observed in any of the experiments. Comparison of 10 independent samples shows that, despite that sometimes the sample sets of structures have relatively large deviations, the derived LM sets vary only slightly, confirming that the sampling is sufficient for the purpose of detecting LMs.

Table 3. Summary of LMs in the lower parts of RNA molecules' landscapes. The composition of LMs (mean values) are given based on their types: pseudoknotted (H, K, L) or pseudoknot-free (N).

ID	LEN	PK	N:H:K:L (% LMs)				# LMs	N:H:K:L with STDEV (# LMs)			
PKB259	57	H	31.1	28.9	14.4	25.6	347.5	108.2±3.0	100.4±5.1	50.0±2.8	88.9±4.9
PKB139	67	H	33.6	50.6	13.6	2.2	4847.1	1628.5±24.9	2452.8±38.3	657.5±14.2	108.3±2.6
PKB173	73	K	13.8	44.6	29.5	12.0	4143.9	573.6±169.0	1848.5±506.4	1224.3±338.1	497.5±153.7
PKB238	84	H	53.3	43.1	2.8	0.8	326.3	174.0±0.0	140.6±7.6	9.0±0.0	2.7±1.4
PKB138	116	H	2.6	70.3	26.9	0.2	1646.9	42.4±2.5	1157.7±12.7	443.5±11.5	3.3±0.8
PKB2	50	H	25.2	47.5	18.6	8.6	4257.6	1072.7±2.5	2024.3±20.5	793.2±9.7	367.4±7.7
PKB49	30	H	27.2	62.2	4.4	6.2	113.8	31.0±0.0	70.8±1.7	5.0±1.0	7.0±0.0
PKB52	52	H	18.7	59.5	10.6	11.2	439.0	82.0±0.0	261.1±3.0	46.6±0.5	49.3±1.2
PKB67	30	H	40.9	59.1	0.0	0.0	22.0	9.0±0.0	13.0±0.0	0.0±0.0	0.0±0.0
PKB70	55	H	21.3	65.1	6.3	7.3	482.5	103.0±0.0	314.2±1.8	30.3±0.6	35.0±0.0
PKB71	108	L	1.2	0.3	98.4	0.0	3860.0	47.5±2.1	11.8±1.9	3799.5±47.0	1.2±0.7
PDB_00213	101	N	34.5	36.3	29.1	0.1	1350.2	466.0±34.9	489.8±44.2	392.9±53.7	1.5±0.7
PDB_00542	126	N	99.4	0.5	0.0	0.0	4605.6	4579.8±72.2	24.3±1.3	1.5±0.7	0.0±0.0
PDB_00702	94	N	22.8	1.0	76.1	0.0	3708.9	846.8±3.2	38.0±1.6	2824.1±6.4	0.0±0.0
PDB_00924	86	N	96.7	0.0	3.3	0.0	276.0	267.0±0.0	0.0±0.0	9.0±0.0	0.0±0.0
PDB_01042	34	N	66.7	33.3	0.0	0.0	12.0	8.0±0.0	4.0±0.0	0.0±0.0	0.0±0.0
RFA_00398	54	N	81.1	12.9	6.0	0.0	443.4	359.8±36.3	57.2±14.6	26.4±6.5	0.0±0.0
SRP_00005	90	N	41.7	54.0	1.3	3.1	4560.3	1902.9±29.8	2460.3±33.5	57.5±2.0	139.6±6.2
SRP_00094	91	N	40.7	57.5	0.2	1.5	9004.0	3668.9±41.2	5178.5±85.7	18.3±1.3	138.3±8.4
SRP_00194	81	N	72.2	27.8	0.0	0.0	1230.1	887.8±1.4	342.3±1.6	0.0±0.0	0.0±0.0
SRP_00284	87	N	89.4	10.6	0.0	0.0	4362.4	3899.6±99.6	461.0±15.6	1.8±0.9	0.0±0.0
TMR_00272	102	N	76.0	23.4	0.4	0.2	2959.7	2248.7±42.0	694.0±23.0	10.5±2.1	6.5±2.2
Bsu	42	N/H	34.5	56.0	6.7	2.9	139.3	48.0±0.0	78.0±2.7	9.3±6.0	4.0±0.0

Table 4. Summary of distinct `gfold`-sampling structures in the lower parts of RNA molecules' landscapes. The composition of structures (mean values) are given based on their types: pseudoknotted (H, K, L) or pseudoknot-free (N).

ID	LEN	PK	N:H:K:L (% Structures)				# Structures	N:H:K:L with STDEV (# Structures)			
PKB259	57	H	69.8	23.3	1.5	5.5	18572.0	12956.3±132.8	4318.7±68.1	276.0±14.3	1021.0±21.4
PKB139	67	H	48.6	46.7	3.1	1.6	112326.3	54563.0±1955.0	52418.1±1630.0	3499.0±113.3	1846.2±65.7
PKB173	73	K	26.0	32.0	29.7	12.3	59852.8	15565.5±3822.0	19125.5±6158.1	17788.2±5380.2	7373.6±2105.4
PKB238	84	H	90.5	6.3	2.9	0.4	46804.2	42336.7±122.2	2932.7±42.6	1368.8±37.1	166.0±8.7
PKB138	116	H	1.3	92.2	6.5	0.0	134964.9	1780.1±33.6	124373.0±188.9	8807.0±85.4	4.8±2.1
PKB2	50	H	34.2	56.6	7.7	1.5	146286.6	50075.1±1966.5	82734.5±2863.5	11310.3±475.1	2166.7±106.8
PKB49	30	H	15.9	82.6	0.4	1.1	5747.5	913.7±195.2	4748.8±849.5	23.7±5.0	61.3±15.6
PKB52	52	H	21.6	65.6	7.2	5.6	26921.1	5810.2±525.0	17655.8±1579.1	1936.5±187.9	1518.6±162.7
PKB67	30	H	52.5	47.5	0.0	0.0	590.0	310.0±0.0	280.0±0.0	0.0±0.0	0.0±0.0
PKB70	55	H	18.2	79.8	1.2	0.8	44393.6	8066.4±163.8	35441.9±635.3	517.5±10.4	367.8±15.7
PKB71	108	L	0.2	0.0	99.8	0.0	193679.6	335.0±12.9	4.1±1.4	193340.2±3873.7	0.3±0.5
PDB_00213	101	N	67.7	29.2	3.1	0.0	63012.5	42633.9±5076.2	18402.7±2380.1	1975.5±318.8	0.4±0.5
PDB_00542	126	N	100.0	0.0	0.0	0.0	148861.8	148813.8±3062.5	46.6±7.1	1.4±1.1	0.0±0.0
PDB_00702	94	N	23.1	0.0	76.9	0.0	241373.1	55672.1±481.9	45.1±4.4	185655.9±1640.0	0.0±0.0
PDB_00924	86	N	99.9	0.0	0.1	0.0	67393.1	67339.0±1480.4	0.0±0.0	54.1±5.1	0.0±0.0
PDB_01042	34	N	100.0	0.0	0.0	0.0	2920.8	2920.8±199.2	0.0±0.0	0.0±0.0	0.0±0.0
RFA_00398	54	N	99.3	0.6	0.1	0.0	25871.1	25680.5±8056.6	165.9±83.2	24.7±14.2	0.0±0.0
SRP_00005	90	N	79.7	20.0	0.0	0.3	134680.9	107326.9±2278.2	26950.3±682.0	59.6±7.7	344.1±20.1
SRP_00094	91	N	78.0	21.9	0.0	0.1	179417.9	139882.2±2179.1	39361.2±858.0	6.1±2.6	168.4±11.2
SRP_00194	81	N	98.9	1.1	0.0	0.0	133245.2	131767.6±3544.4	1477.6±73.8	0.0±0.0	0.0±0.0
SRP_00284	87	N	98.3	1.7	0.0	0.0	122341.4	120219.1±4052.3	2120.8±96.4	1.5±1.2	0.0±0.0
TMR_00272	102	N	93.0	7.0	0.0	0.0	83103.6	77262.7±2144.9	5832.6±204.1	5.4±2.1	2.9±0.8
Bsu	42	N/H	61.7	37.4	0.4	0.4	4841.0	2989.2±180.4	1812.6±110.9	19.6±1.2	19.6±1.2

5.2 Robustness of BHG-approach in estimating saddle heights

As shown in Table 3, the LM sets are fairly stable when sufficiently large sets are sampled. Of course, the LM sets obtained from independent samplings are usually not identical since the high energy LMs grow exponentially in number and thus cannot be exhaustively collected in practise. The BHGs constructed based on these LM sets therefore will differ in vertex sets, edge sets and the weights (saddle heights) on edges. We therefore show that these BHGs nevertheless agree to high accuracy on the low-energy LMs, *and* the edges between them. As a consequence, the estimations of saddle heights between them are also robust.

To this end, we first compute BHGs based on 10 independent samples for a given RNA sequence, collect the set of the common LMs in these BHGs and then evaluate the standard deviations of saddle heights between all these pairs of common LMs. The average standard deviations are reported in Table 5. Note that the number of common LMs is different from the number of LMs generated with `RNAlocmin` in Table 3. This is because the heuristic algorithm constructing the BHGs first selects the non-shallow LMs from the initial LM set generated from `RNAlocmin` and then iteratively expands this set of non-shallow LMs by adding intermediate LMs detected in the path searching procedure. For three examples PDB_00542, PDB_00702, and SRP_00005, the evaluation described above is computationally infeasible due to the large numbers (more than 13 thousands) of the common LMs in their BHGs. Given a set of K LMs, evaluating saddle heights between all pairs of these LMs requires $O(K^3)$ time using a variant of Dijkstra's algorithm to detect the corresponding shortest min-max paths. Instead of the entire set, we therefore evaluate only the lowest 1000 common LMs.

Given that the averaged deviations are less than 0.26 kcal/mol, we conclude that our method in estimating saddle heights is robust.

Table 5. Summary of the saddle heights estimated based on 10 independent samples. It shows the average standard deviations (STDEV) of the saddle heights between all (except 3 large examples) and for the subset of the lowest 1000 common LMs. The saddle heights are evaluated in units of kcal/mol.

ID	# LMs in common	STDEV (on average) All	STDEV (on average) Lowest 1000
PKB259	442	0.047	0.047
PKB139	6256	0.12	0.017
PKB173	13786	0.116	0.045
PKB238	7246	0.169	0.129
PKB138	5682	0.134	0.064
PKB2	3953	0.04	0.014
PKB49	1154	0.054	0.048
PKB52	5158	0.153	0.046
PKB67	624	0.007	0.007
PKB70	3199	0.149	0.064
PKB71	2681	0.124	0.123
PDB_00213	4136	0.169	0.049
PDB_00542	20430	NA	0.07
PDB_00702	29270	NA	0.031
PDB_00924	5670	0.252	0.122
PDB_01042	969	0.112	0.112
RFA_00398	1187	0.108	0.1
SRP_00005	13670	NA	0.041
SRP_00094	11769	0.174	0.018
SRP_00194	1190	0.141	0.119
SRP_00284	8135	0.102	0.031
TMR_00272	4931	0.133	0.057
Bsu	816	0.114	0.114

6 PART F: SADDLE HEIGHT CHANGES BETWEEN BHG$^\psi$ AND BHG$^\circ$

6.1 Histograms of saddle height changes between BHG$^\psi$ and BHG$^\circ$

In the following, we only consider for RNAs whose ground states are pseudoknot-free. For each of such RNAs, the full BHG$^\psi$ including pseudoknotted LMs and a pruned BHG$^\circ$ from which first all pseudoknotted LMs are removed and then the BHG-adjacency is recomputed using only pseudoknot-free structures along the paths. The re-evaluation may result in the removal of adjacencies from BHG$^\psi$.

Five examples (PDB_00542, PDB_01042, RFA_00398, SRP_00194 and SRP_00284) were not shown given that the differences between BHG$^\psi$ and BHG$^\circ$ are relatively small.

Histograms of saddle height changes between BHG$^\psi$ and BHG$^\circ$ for 8 RNAs are shown in Fig. 9-15. In each example, the x-axes of the top and bottom histograms denote the exact changes (in kcal/mol) and relative changes (in %), respectively. The y-axes denote the corresponding numbers of pseudoknot-free LMs pairs with such saddle changes. The colors of the histograms indicate the types of pseudoknotted structures appear in the energetically optimal paths between LM pairs. For example, the pink color (Type HK) indicates that the energetically optimal paths contain structures with both H-type and K-type pseudoknots. Green (Type N) indicates the simulated paths do not contain any pseudoknotted structures.

6.2 Using `--depth` parameter in `findpath` to improve negative saddle height differences between BHG$^\psi$ and BHG$^\circ$

In general, saddle heights between the LMs in BHG$^\circ$ will increase compared to BHG$^\psi$. In practice, however, the inclusion of additional LMs during the recomputation of the adjacencies can in rare cases lead to an apparent decrease in saddle heights and furthermore negative saddle heights differences between BHG$^\psi$ and BHG$^\circ$. For example, see Fig. 16.

In such cases, the saddle heights in BHG$^\psi$ are overestimated due to the heuristic nature of the `findpath` program (Flamm *et al.*, 2000) used to estimate saddle heights. `Findpath` performes a bounded breadth-first search algorithm that at each depth only keeps the m most promising candidates. The option "–`depth`" with default value 10 is used to specify m and therefore balances speed versus accuracy. As we show in Fig. 17, once we increase the candidate number to 100, all negative saddle height differences are eliminated, see Fig. 16.

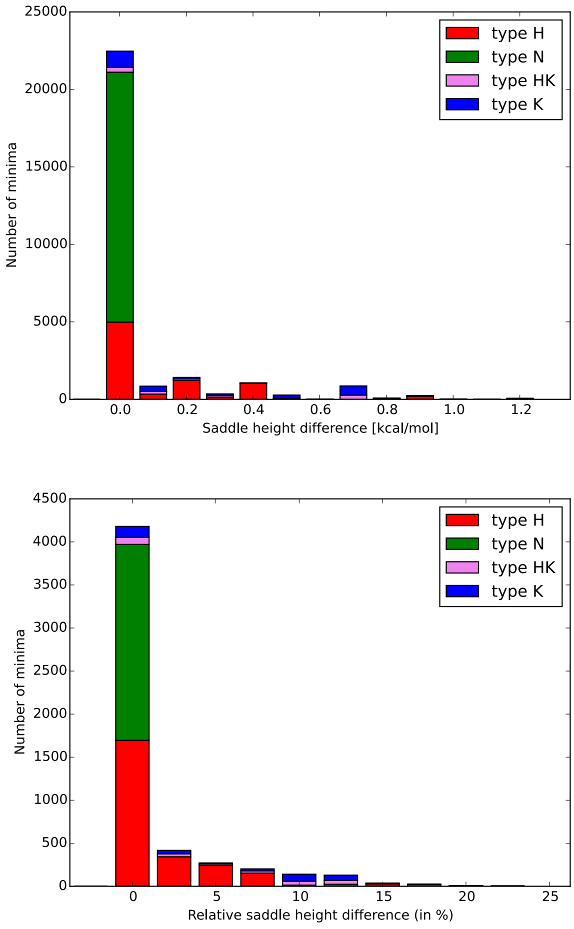

Fig. 9. Histograms of saddle height changes between BHG$^{\psi}$ and BHG$^{\circ}$ of the transcriptional preQ$_1$ riboswitch of *Bacillus subtilis* (Bsu, Suddala *et al.* (2013)). The relevant saddle heights are generated with `findpath` using default parameter `--depth=10`.

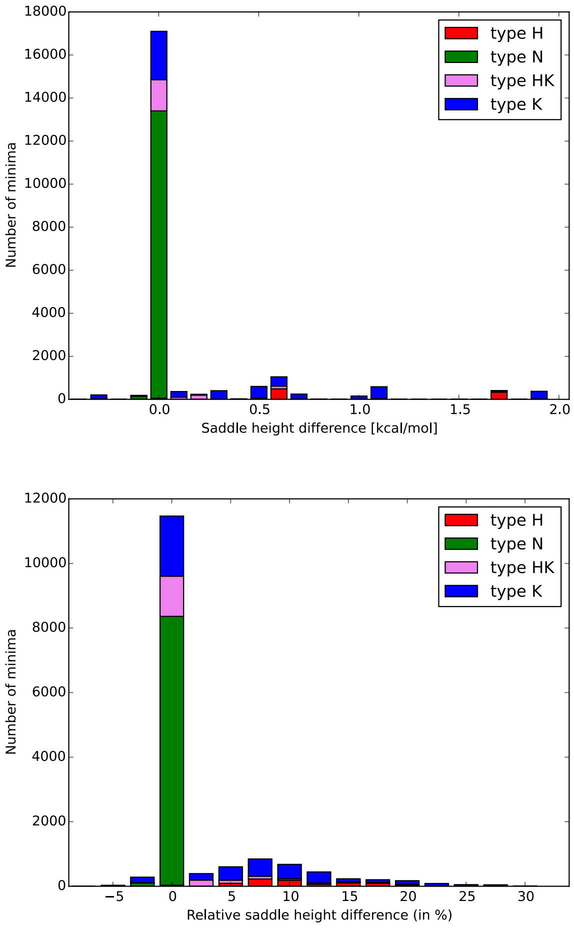

Fig. 10. Histograms of saddle height changes between BHG$^{\psi}$ and BHG$^{\circ}$ of the core encapsidation signal of the *Moloney murine* leukemia virus (PDB_00213, D'Souza *et al.* (2004)). The relevant saddle heights are generated with `findpath` using default parameter `--depth=10`.

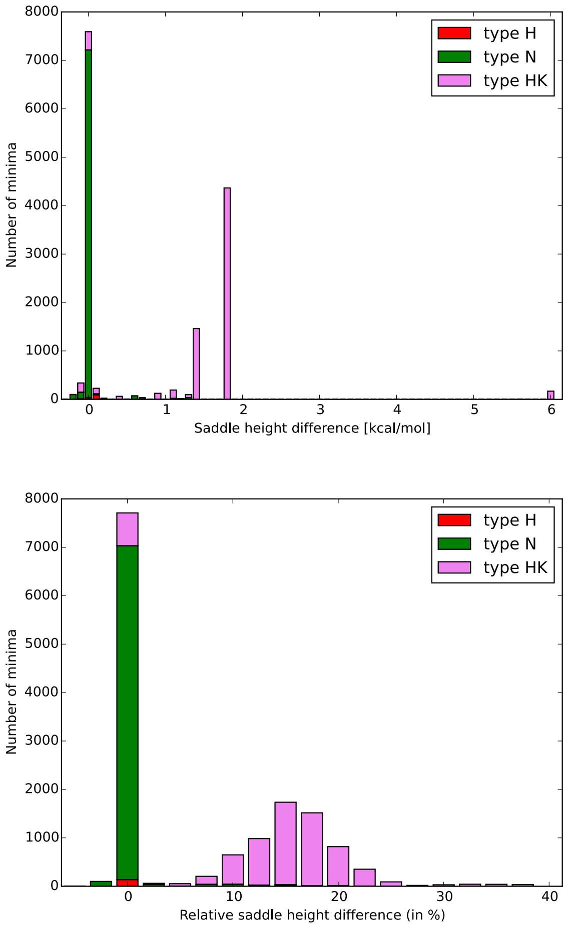

Fig. 11. Histograms of saddle height changes between BHG^{ψ} and BHG° of a signal recognition particle of *M. Jannaschiii* (PDB_00879, Hainzl *et al.* (2005)). The relevant saddle heights are generated with `findpath` using default parameter `--depth=10`.

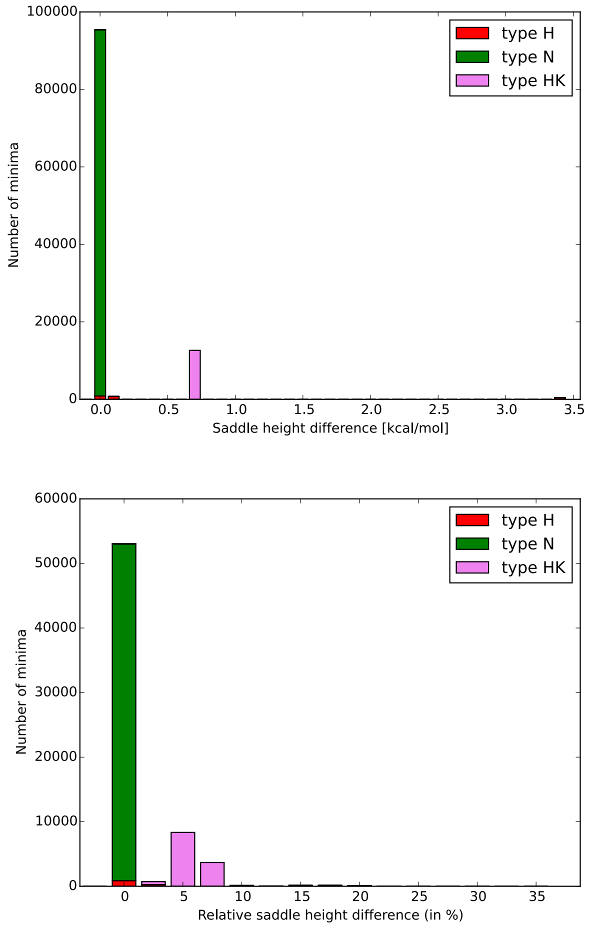

Fig. 12. Histograms of saddle height changes between BHG^{ψ} and BHG° of a synthetic tetraloop-receptor (PDB_00924, Davis *et al.* (2005)). The relevant saddle heights are generated with `findpath` using default parameter `--depth=10`.

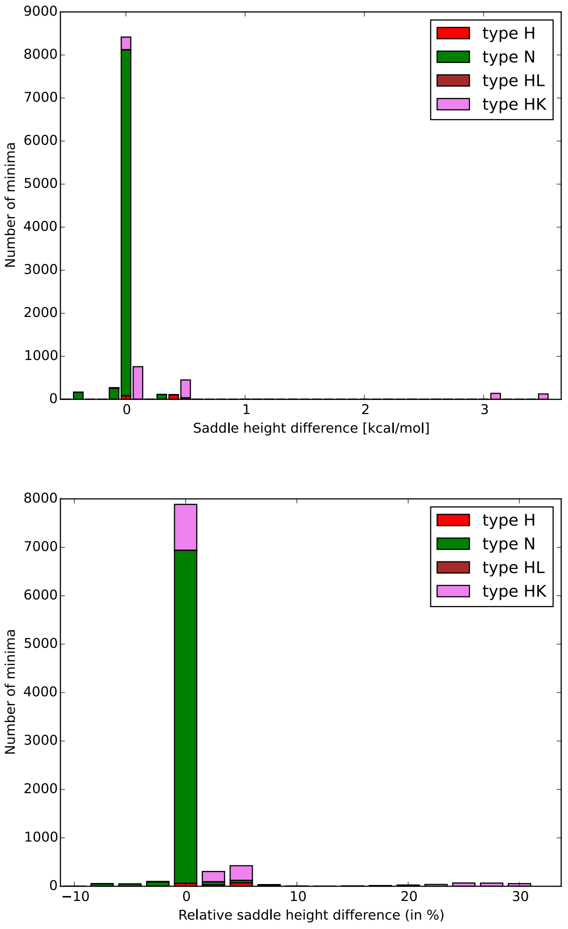

Fig. 13. Histograms of saddle height changes between BHG^{ψ} and BHG° of a signal recognition particle RNA provided in the SRPDB database (SRP_00005, Rosenblad *et al.* (2003)). The relevant saddle heights are generated with `findpath` using default parameter `--depth=10`.

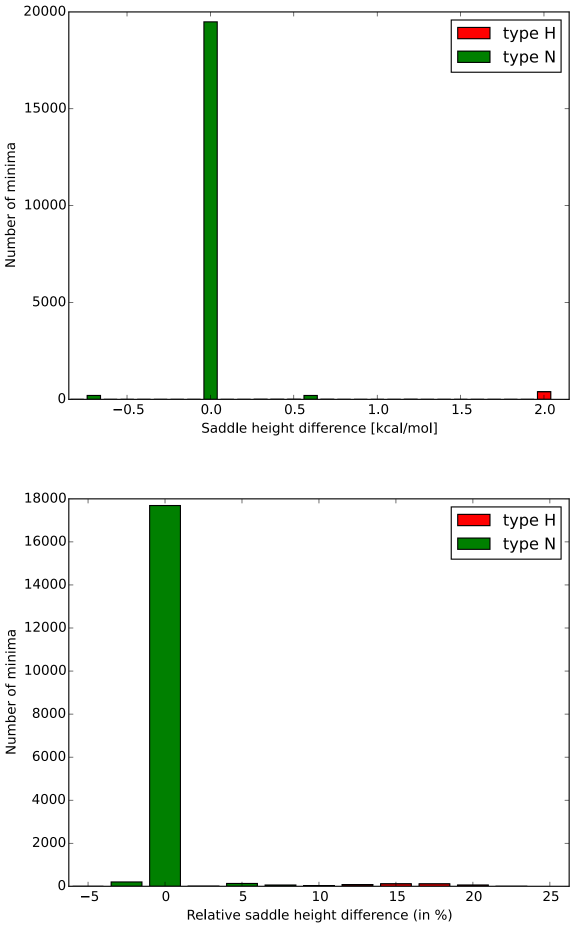

Fig. 14. Histograms of saddle height changes between BHG^{ψ} and BHG° of a signal recognition particle RNA provided in the SRPDB database (SRP_00094, Rosenblad *et al.* (2003)). The relevant saddle heights are generated with `findpath` using default parameter `--depth=10`.

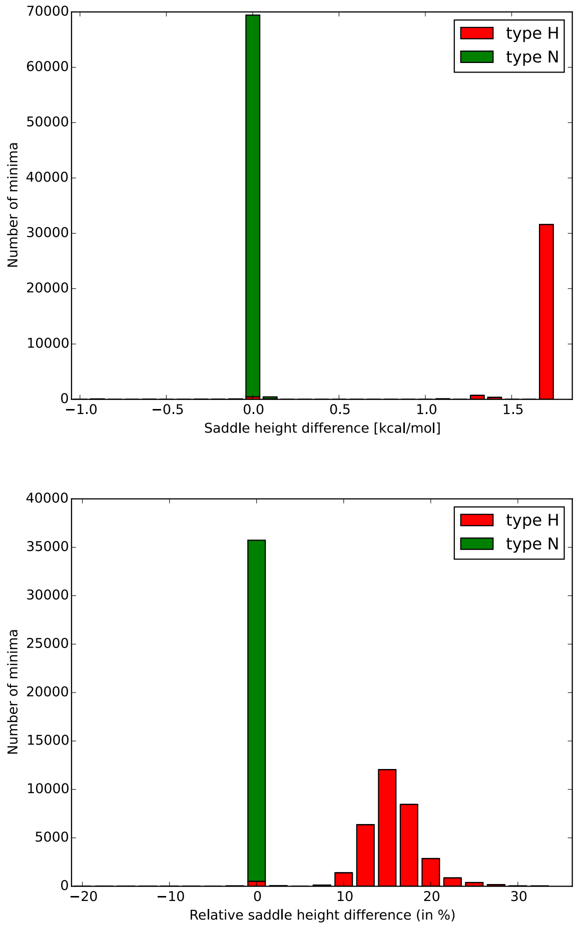

Fig. 15. Histograms of saddle height changes between BHG$^{\psi}$ and BHG$^{\circ}$ of a tmRNA provided in the tmRDB database (TMR_00272, Knudsen *et al.* (2001)). The relevant saddle heights are generated with `findpath` using default parameter `--depth=10`.

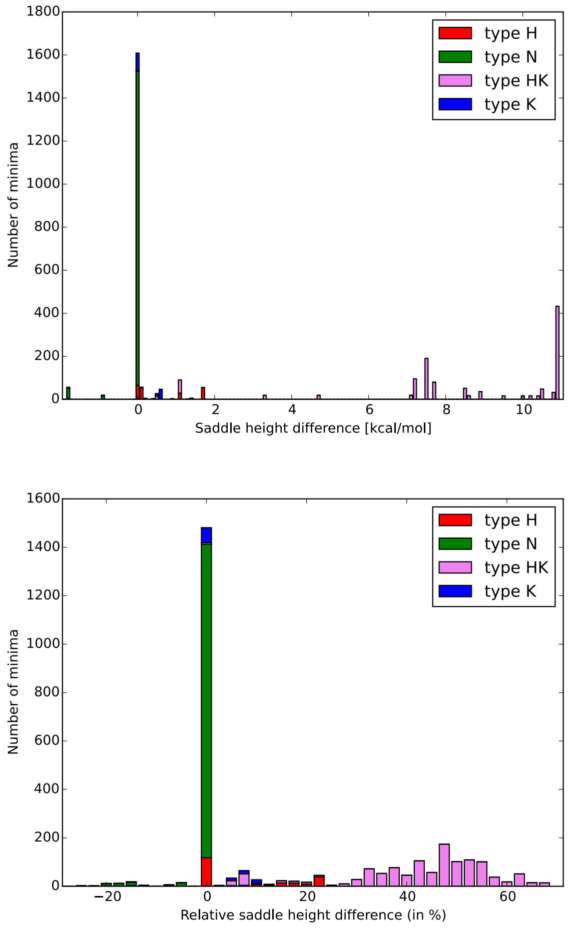

Fig. 16. Histograms of saddle height changes between BHG$^{\psi}$ and BHG$^{\circ}$ of a Ribosomal RNA of *E. coli* (PDB_00702, Merianos *et al.* (2004)). The relevant saddle heights are generated with `findpath` using default parameter `--depth=10`.

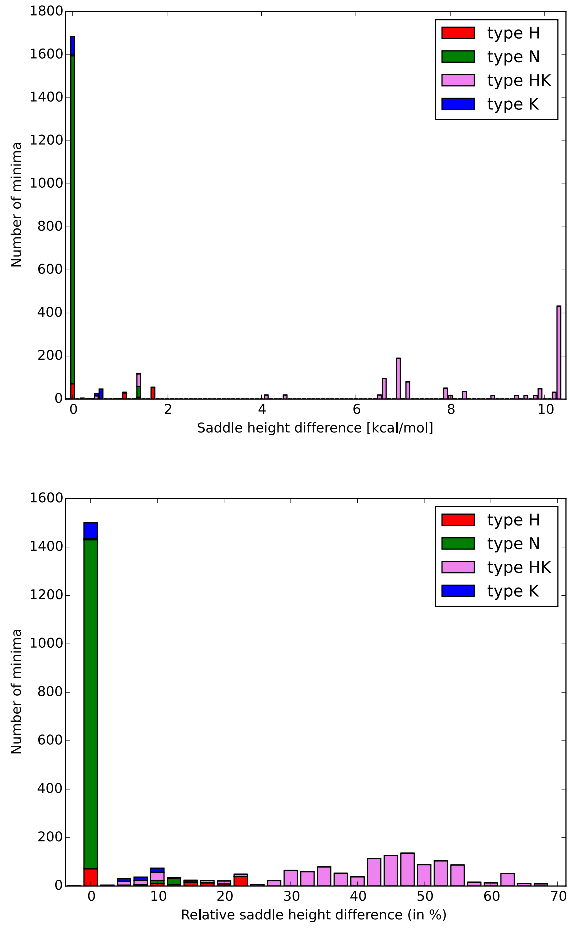

Fig. 17. Histograms of saddle height changes between BHG^{ψ} and BHG° of a Ribosomal RNA of *E. coli* (PDB_00702, Merianos *et al.* (2004)). The relevant saddle heights are generated with `findpath` using parameter `--depth=100`.

7 PART G: FOLDING KINETICS OF RNAS

In the following we only consider for RNAs whose ground states are pseudoknot-free. For each of such RNAs, the full BHG$^\psi$ including pseudoknotted LMs and a pruned BHG$^\circ$ from which first all pseudoknotted LMs are removed and then the BHG-adjacency is recomputed using only pseudoknot-free structures along the paths. The reevaluation may result in the removal of adjacencies from BHG$^\psi$.

Seven examples (PDB_00542, PDB_00924, PDB_01042, RFA_00398, SRP_00194, SRP_00284 and TMR_00272) were not shown given that the differences in kinetics between BHG$^\psi$ and BHG$^\circ$ are relatively small. The kinetics on BHG$^\circ$ and BHG$^\psi$ of three RNAs are shown in the top and bottom of Fig. 12, 13, and 14, respectively. The process was started in the open chain structure and run until convergence to the thermodynamic equilibrium distribution. Dotted vertical line indicates when the simulation reaches its equilibrium. The LMs that appear in both kinetics plots are marked with the same color, otherwise pseudoknot-free and pseudoknotted LMs are marked with black and red, respectively. The sums of the population probabilities of pseudoknot-free and pseudoknotted LMs on BHG$^\psi$ are shown with blue and red broken lines, respectively.

8 PART H: MAXIMUM LIKELIHOOD CRITERION WITHIN UPPER TIME LIMIT T

Given a trajectory $U = (s_0, t_0, s_1, t_1, \ldots, s_{k-1}, t_{k-1}, s_k)$ that the molecule started in s_0, where it stayed for time t_0, then transitioned to s_1, where it stayed for time t_1, and so on until eventually it reached s_k, where it remained until time T. The likelihood of such a trajectory U is

$$\Pi_{i=0}^{k-1}\left(\lambda_{s_i} \cdot e^{-\lambda_{s_i} t_i} \cdot P_{s_i,s_{i+1}}\right) \cdot e^{-\lambda_{s_k}(T-\sum t_i)} \qquad (1)$$

when $\sum t_i \leq T$ and 0 otherwise. In our cases, we have $\lambda_{s_i} = \sum_{s_j} r_{s_i s_j}$ and $P_{s_i,s_{i+1}} = \frac{r_{s_i s_{i+1}}}{\lambda_{s_i}}$. Therefore, equation reduces to

$$\Pi_{i=0}^{k-1} r_{s_i s_{i+1}} \cdot e^{-\left(\sum_{i=0}^{k-1} \lambda_{s_i} t_i + \lambda_{s_k}(T-\sum t_i)\right)}. \qquad (2)$$

We compute the optimal paths for two cases $T = 0$ and $T = 10^{11}$ for the SV11 sequence shown in Fig. 21. Notice here when $T = 0$, all t_i has to be 0 as well. Eqn. 2 furthermore reduces to $\Pi_{i=0}^{k-1} r_{s_i s_{i+1}}$ Therefore the Criterion C can be seen as a special case of the Criterion B when $T = 0$.

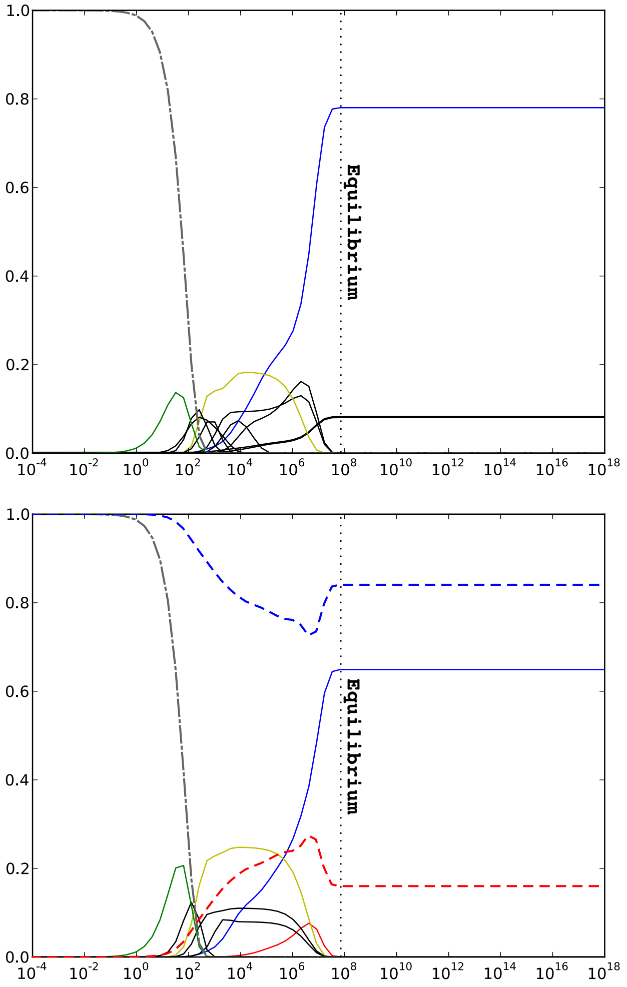

Fig. 18. Folding kinetics of the core encapsidation signal of the *Moloney murine* leukemia virus (PDB_00213, D'Souza *et al.* (2004)).

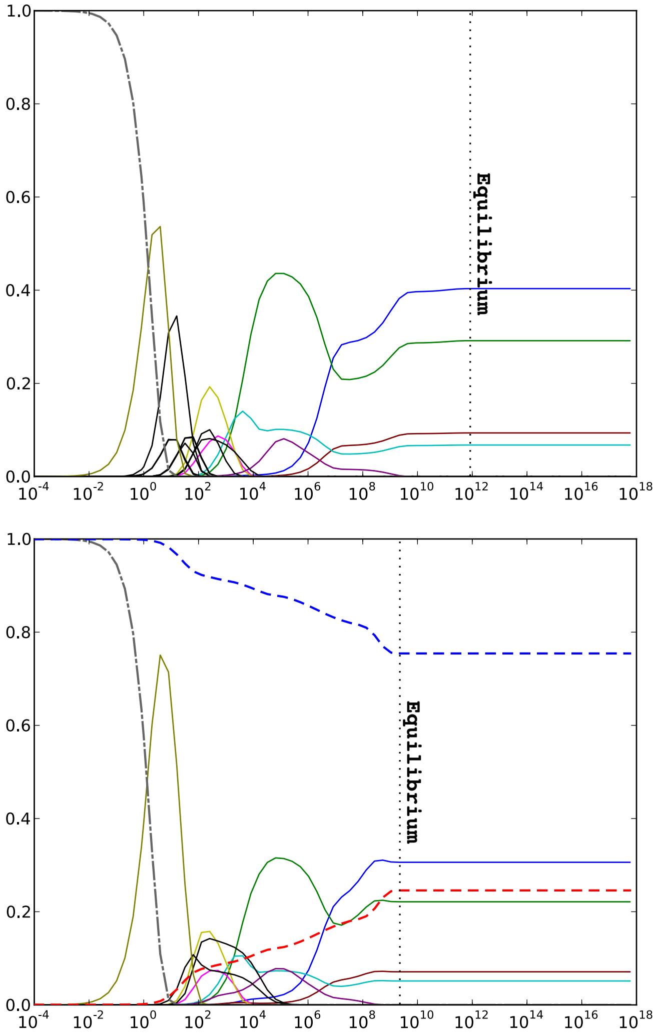

Fig. 19. Folding kinetics of a signal recognition particle RNA of *M. Jannaschiii* (PDB_00879, Hainzl *et al.* (2005)).

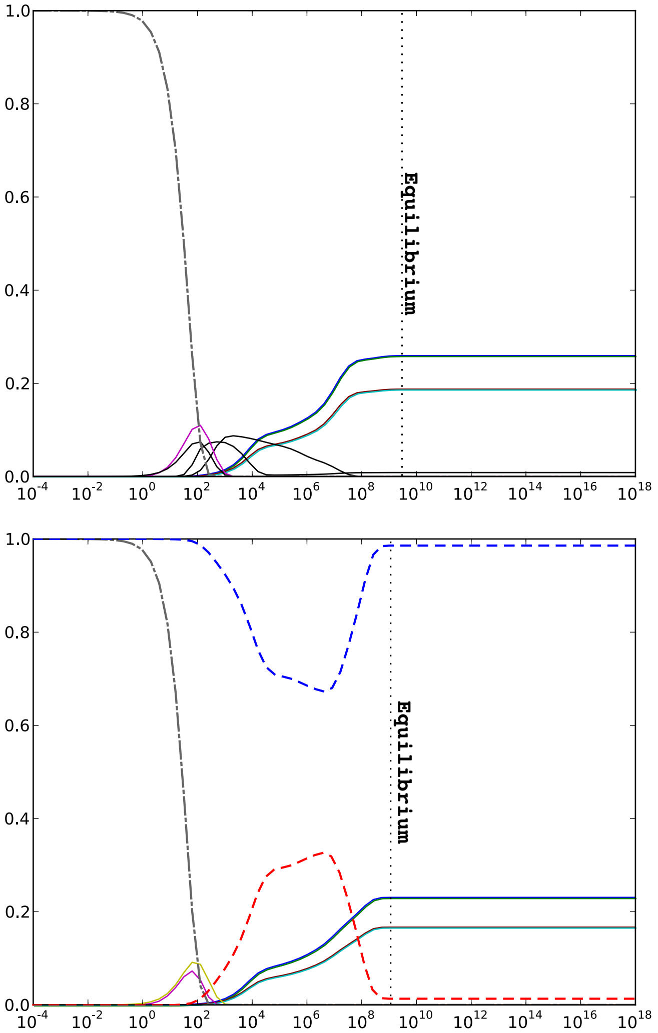

Fig. 20. Folding kinetics of a signal recognition particle RNA provided in the SRPDB database (SRP_00005, Rosenblad *et al.* (2003)).

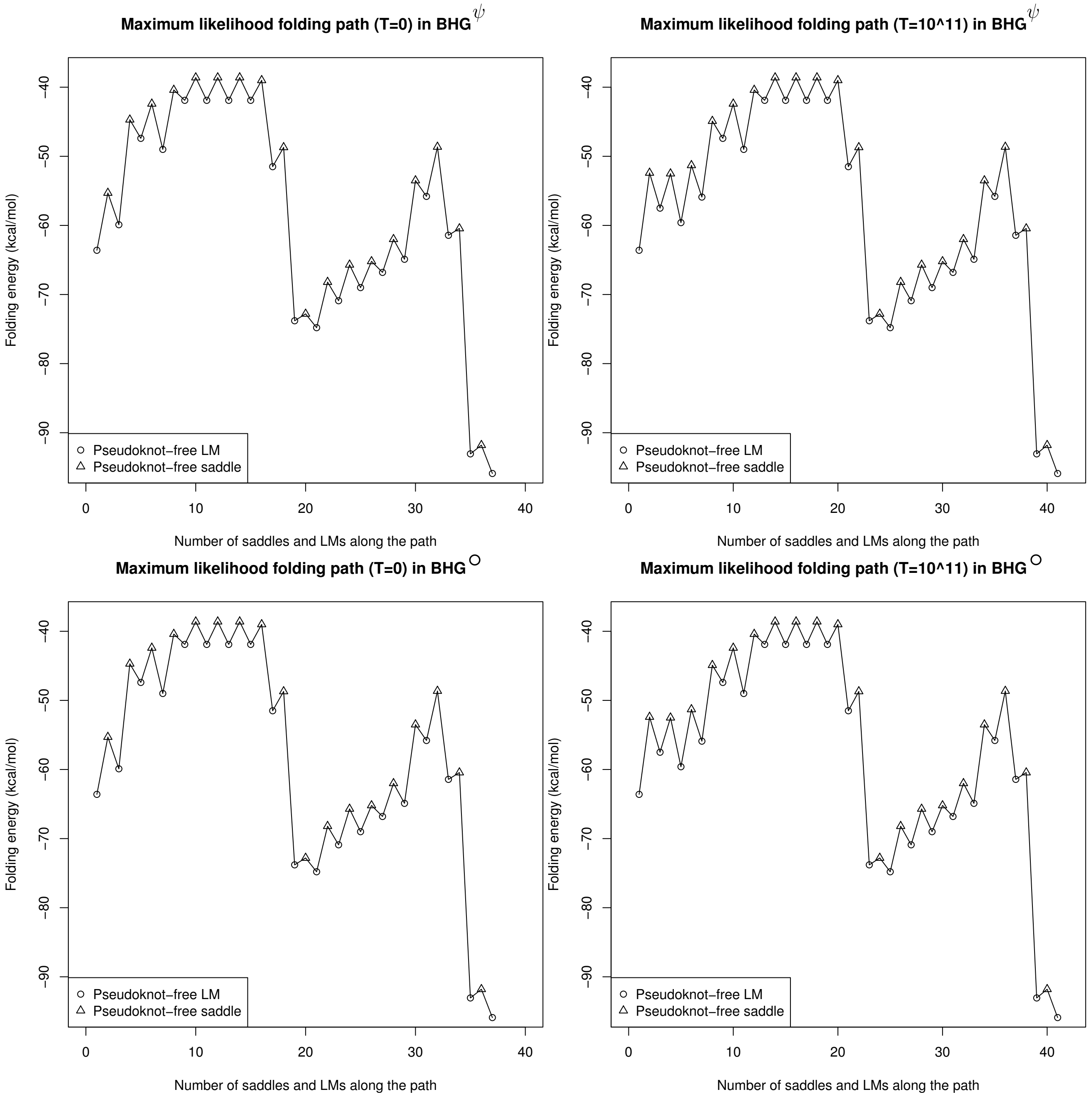

Fig. 21. Maximum likelihood criterion with time limit $T = 0$ (Left) and $T = 10^{11}$ (Right) for the SV11 sequence.

REFERENCES

Akutsu, T. (2000) Dynamic programming algorithms for RNA secondary structure prediction with pseudoknots. *Discrete Appl Math,* **104**, 45–62.

Al-Mohy, A. & Higham, N. (2009) A new scaling and squaring algorithm for the matrix exponential. *SIAM J. Matrix Anal.,* **31**, 970–989.

Andronescu, M., Bereg, V., Hoos, H. & Condon, A. (2008) RNA STRAND: The RNA Secondary Structure and Statistical Analysis Database. *BMC Bioinformatics,* **9**.

Bon, M., Vernizzi, G., Orland, H. & Zee, A. (2008) Topological classification of rna structures. *J. Mol. Biol.,* **379** (4), 900–911.

Davis, J., Tonelli, M., Scott, L., Jaeger, L., Williamson, J. & Butcher, S. (2005) RNA helical packing in solution: NMR structure of a 30 kDa GAAA tetraloop-receptor complex. *J. Mol. Biol.,* **351** (2), 371–382.

D'Souza, V., Dey, A., Habib, D. & Summers, M. (2004) NMR structure of the 101-nucleotide core encapsidation signal of the Moloney murine leukemia virus. *J. Mol. Biol.,* **19** (2), 427–442.

Flamm, C., Fontana, W., Hofacker, I. & Schuster, P. (2000) RNA folding kinetics at elementary step resolution. *RNA,* **6**, 325–338.

Flamm, C., Hofacker, I., Stadler, P. & Wolfinger, W. (2002) Barrier trees of degenerate landscapes. *Z. Phys. Chem.,* **216**, 155–173.

Flamm, C., Hofacker, I. L., Maurer-Stroh, S., Stadler, P. F. & Zehl, M. (2000) Design of multi-stable RNA molecules. *RNA,* **7**, 254–265.

Hainzl, T., Huang, S. & Sauer-Eriksson, A. (2005) Structural insights into SRP RNA: an induced fit mechanism for SRP assembly. *RNA,* **11** (7), 1043–1050.

Han, K., Lee, Y. & W., K. (2002) PseudoViewer: automatic visualization of RNA pseudoknots. *Bioinformatics,* **18** (Suppl 1), 321–328.

Hofacker, I. L., Fontana, W., Stadler, P. F., Bonhoeffer, S., Tacker, M. & Schuster, P. (1994) Fast folding and comparison of RNA secondary structures. *Monatsh. Chem.,* **125**, 167–188.

Isambert, H. & Siggia, E. D. (2000) Modeling RNA folding paths with pseudoknots: Application to hepatitis delta virus ribozyme. *Proc. Natl. Acad. Sci. USA,* **97**, 6515–6520.

Knudsen, B., Wower, J., Zwieb, C. & Gorodkin, J. (2001) tmRDB (tmRNA database). *Nucleic Acids Res.,* **29** (1), 171–172.

Lyngso, R. & Pedersen, C. (2000) RNA pseudoknot prediction in energy-based models. *J. Comput. Biol.,* **7**, 409–427.

Mathews, D., Sabina, J., Zuker, M. & Turner, D. H. (1999) Expanded sequence dependence of thermodynamic parameters improves prediction of RNA secondary structure. *J. Mol. Biol.,* **288**, 911–940.

Maňuch, J., Thachuk, C., Stacho, L. & Condon, A. (2011) NP-completeness of the energy barrier problem without pseudoknots and temporary arcs. *Natural Computing,* **10** (1), 391–405.

Merianos, H., Wang, J. & Moore, P. (2004) The structure of a ribosomal protein S8/spc operon mRNA complex. *RNA,* **10** (6), 954–964.

Reidys, C., Huang, F., Andersen, J., Penner, R., Stadler, P. & Nebel, M. (2011) Topology and prediction of rna pseudoknots. *Bioinformatics,* **27** (8), 1076–1085.

Reidys, C. M. & Stadler, P. F. (2002) Combinatorial landscapes. *SIAM Review,* **44**, 3–54.

Rosenblad, M., Gorodkin, J., Knudsen, B., Zwieb, C. & Samuelsson, T. (2003) SRPDB: Signal Recognition Particle Database. *Nucleic Acids Res.,* **1** (31), 363–364.

Sibani, P., van der Pas, R. & Schön, J. C. (1999) The lid method for exhaustive exploration of metastable states of complex systems. *Computer Physics Communications,* **116**, 17–27.

Suddala, K. C., Rinaldi, A. J., Feng, J., Mustoe, A. M., Eichhorn, C. D., Liberman, J. A., Wedekind, J. E., Al-Hashimi, H. M., Brooks, C. L. & Walter, N. G. (2013) Single transcriptional and translational preq1 riboswitches adopt similar pre-folded ensembles that follow distinct folding pathways into the same ligand-bound structure. *Nucleic Acids Res,* **41** (22), 10462–10475.

Taufer, M., Licon, A., Araiza, R., Mireles, D., van Batenburg, F., Gultyaev, A. & Leung, M. (2009) Pseudobase++: an extension of pseudobase for easy searching, formatting and visualization of pseudoknots. *Nucleic Acids Res.,* **37** (Database-Issue), 127–135.

Wolfinger, M. T., Svrcek-Seiler, W. A., Flamm, C., Hofacker, I. L. & Stadler, P. F. (2004) Exact folding dynamics of RNA secondary structures. *J. Phys. A: Math. Gen.,* **37**, 4731–4741.
